# A new immune signature for survival prediction and immune checkpoint molecules in lung adenocarcinoma

**DOI:** 10.1186/s12967-020-02286-z

**Published:** 2020-03-06

**Authors:** Dina Guo, Mian Wang, Zhihong Shen, Jiaona Zhu

**Affiliations:** Department of Infectious Diseases, Ningbo Yinzhou No.2 Hospital, Ningbo, 315100 Zhejiang China

**Keywords:** Immune, Signature, Prognosis, Prediction, Immune checkpoint molecules, Lung adenocarcinoma

## Abstract

**Background:**

Lung adenocarcinoma (LUAD) is the most frequent subtype of lung cancer. The prognostic signature could be reliable to stratify LUAD patients according to risk, which helps the management of the systematic treatments. In this study, a systematic and reliable immune signature was performed to estimate the prognostic stratification in LUAD.

**Methods:**

The profiles of immune-related genes for patients with LUAD were used as one TCGA training set: n = 494, other validation set 1: n = 226 and validation set 2: n = 398. Univariate Cox survival analysis was used to identify the candidate immune-related genes from each cohort. Then, the immune signature was developed and validated in the training and validation sets.

**Results:**

In this study, functional analysis showed that immune-related genes involved in immune regulation and MAPK signaling pathway. A prognostic signature based on 10 immune-related genes was established in the training set and patients were divided into high-risk and low-risk groups. Our 10 immune-related gene signature was significantly related to worse survival, especially during early-stage tumors. Further stratification analyses revealed that this 10 immune-related gene signature was still an effective tool for predicting prognosis in smoking or nonsmoking patients, patients with KRAS mutation or KRAS wild-type, and patients with EGFR mutation or EGFR wild-type. Our signature was negatively correlated with B cell, CD4+ T cell, CD8+ T cell, neutrophil, dendritic cell (DC), and macrophage immune infiltration, and immune checkpoint molecules PD-1 and CTLA-4 (*P *< 0.05).

**Conclusions:**

These findings suggested that our signature was a promising biomarker for prognosis prediction and can facilitate the management of immunotherapy in LUAD.

## Background

Lung carcinoma is the most frequent human malignant neoplasm and the highest cause of cancer-related deaths worldwide [1]. Despite recent advances in surgery, chemotherapy, radiotherapy, targeted therapy and immunotherapy, the 5-year survival rate of patients with non-small cell lung cancer (NSCLC) remains poor [2, 3]. NSCLC, accounting for approximately 85% of lung cancer cases, consists of two common histological subtypes: lung adenocarcinoma (LUAD) and squamous cell carcinoma (LUSC) [4]. Increasing evidence suggests the well-known biological diversity of LUAD and LUSC. For example, epidermal growth factor receptor (EGFR) mutations are more frequent in LUAD than in LUSC [5]. LUAD is the most common subtype of NSCLC [6].

The immune system has been reported to play an essential role in the development and progression of cancer [7, 8]. Tumor immunotherapy as an important driver of personalized medicine harness the anti-tumor effects of the immune system to obtain a durable cure with minimal toxicity [9, 10]. In recent years, the immune checkpoint proteins such as cytotoxic T-lymphocyte antigen 4 (CTLA-4) or the programmed cell death ligand 1/protein 1 pathway (PD-L1/PD-1) have been used as crucial targets for immunotherapy in many cancers, including LUAD [11, 12]. For example, PD-L1 expression is a predictive biomarker for worse prognosis of NSCLC and the probability of clinical benefit from immune-modulating drugs is greater in NSCLC patients expressing PD-L1 [13, 14]. Most studies on immune-related genes such as PD-L1, CD8A, and CD4 expression have been conducted in NSCLC [15–17]. Because the biological differences among the various histological subtypes might have a different therapeutic influence. Moreover, the molecular characteristics describing tumor-immune effects still remain largely unclear in LUAD. Therefore, we focused exclusively on the study of LUAD to further explore the prognostic and predictive significance of the immune-related genes.

In our present work, multiple cohorts were utilized to construct and validate a novel immune prognostic signature for the stratification of LUAD. This study provided more in-depth insight into the prognostic stratification of patients with LUAD as well as provided a tumor-immune interaction with great promise for the therapeutic interventions of LUAD.

## Materials and methods

### Study samples

The raw expression data (Workflow Type: HTSeq-Counts) for LUAD were enrolled from Genomic Data Commons Data Portal, which fulfilled the approval of the project by the consortium. The Cancer Genome Atlas (TCGA) data were normalized by the Trimmed Mean of M-values (TMM) method and the mean expression levels with ≤ 1 were excluded. Then, the expression data were transformed with log2. For the prognostic information, patients with incomplete follow-up time were excluded. Finally, 494 cases with LUAD had sufficient survival data recorded. The Gene Expression Omnibus (GEO) datasets by microarray were used in the patients with LUAD. IRON normalization was performed on the GSE72094, with log2 expression. The GSE31210 data were normalized by the MAS5 algorithm and the expression data were transformed with log2. The cases with insufficient follow-up time were excluded. Finally, 226 patients from GSE31210 (validation set 1) and 398 cases from GSE72094 (validation set 2) were included.

In this study, TCGA data was applied as a training set. The remaining two cohorts were applied as two validation sets. Overall survival (OS) was estimated from the date of the study enrollment to the recorded date of death of any cause or the last follow-up time. The clinicopathological characteristics of LUAD patients from the training and validation sets are listed in Table 1.

**Table 1:** Clinicopathological characteristics of LUAD patients from the training and validation sets

Characteristics	Training set (n = 494)		Validation set 1 (n = 226)		Validation set 2 (n = 398)	
	Number of cases	%	Number of cases	%	Number of cases	%
Median	66 (33–88)		61 (30–76)		70 (38–89)	
Age (years)						
≥ 65	274	55.5	62	27.4	291	73.1
< 65	220	44.5	164	72.6	107	26.9
Gender						
Male	228	46.2	105	46.5	176	44.2
Female	266	53.8	121	53.5	222	55.8
Stage						
Stage 3–4	106	21.8	0	0	72	18.3
Stage 1–2	380	78.2	226	100	321	81.7
Smoking						
Yes	411	85.6	111	49.1	300	90.6
No	69	14.4	115	50.9	31	9.4
KRAS mutation						
Mutation			20	8.8	139	34.9
Wild-type			206	91.1	259	65.1
EGFR mutation						
Mutation			127	56.2	41	10.3
Wild-type			99	43.8	357	89.7
T						
T 3–4	64	13				
T 1–2	427	87				
M						
Positive	25	7.1				
Negative	325	92.9				
N						
Positive	164	34				
Negative	318	66				

### The functional analysis of immune genes

We applied the Immunology Database and Analysis Portal (ImmPort) database to select immune-related genes in immunology research [18, 19]. Using univariate Cox regression analysis, 299 survival-related genes with *P *< 0.05 were found in the TCGA training set (Additional file 1: Table S1), where further analyzed for functional enrichment analysis. The functional analysis was conducted using clusterProfiler package [20] for gene ontology (GO) terms and the Kyoto Encyclopedia of Genes and Genomes (KEGG) pathways.

### Development of the immune-related gene prognostic signature

Immune-related genes were obtained from the ImmPort database (https://immport.niaid.nih.gov) (Additional file 2: Table S2). We performed the following process to establish the immune signature. First, univariate Cox regression analysis with *P *< 0.05 was conducted to find the potential immune-related prognostic genes. Three data sets were used to get overlapping genes, which can increase the credibility of the potential immune-related prognostic genes, the final 52 overlapping survival-related immune genes were found (Additional file 3: Table S3 and Additional file 4: Figure S1). Second, to achieve the final immune-related prognostic genes in our model, multivariate Cox regression analysis based on the two-step method was finally carried out for these 52 candidate immune-related genes in the TCGA training set. Finally, 10 immune-related genes were identified from the TCGA cohort in the present model (Additional file 5: Table S4).

### Tumor-infiltrating immune cells

TIMER was applied to estimate the abundance of tumor-infiltrating immune cells in the tumor microenvironment (TME) (http://cistrome.dfci.harvard.edu/TIMER/), including six immune cell types: B cell, CD4 T cell, CD8 T cell, neutrophil, macrophage, and dendritic cell (DC). We investigated whether our signature played a role in immune infiltration.

### Statistical analysis

Spearman’s correlation coefficient was used to assess the association between risk score and tumor-infiltrating immune cells and immune checkpoint molecules. The cut-off value was determined based on the median value in the training set. Patients were divided into low-risk group and high-risk group. To examine significant differences of between the low-risk group and the corresponding high-risk group, Kaplan–Meier survival methods with the log-rank test were applied. To estimate the predictive accuracy of the immune-related gene signature, time-dependent receiver-operating characteristic (ROC) method was conducted in all datasets. Univariate and multivariate survival analyses were conducted using the Cox proportional hazard model to determine whether there was a significant relationship between the immune signature and the survival of patients with LUAD, along with the clinicopathological variables in the training and validation sets. Subgroup analyses were also carried out according to the stratification of age, gender, smoking status, tumor stage, KRAS mutation, and EGFR mutation status. All data were analyzed by using R software (version 3.5.1; R Foundation for Statistical Computing, Vienna, Austria).

## Results

### The molecular mechanism of immune-related genes

**Fig. 1:**
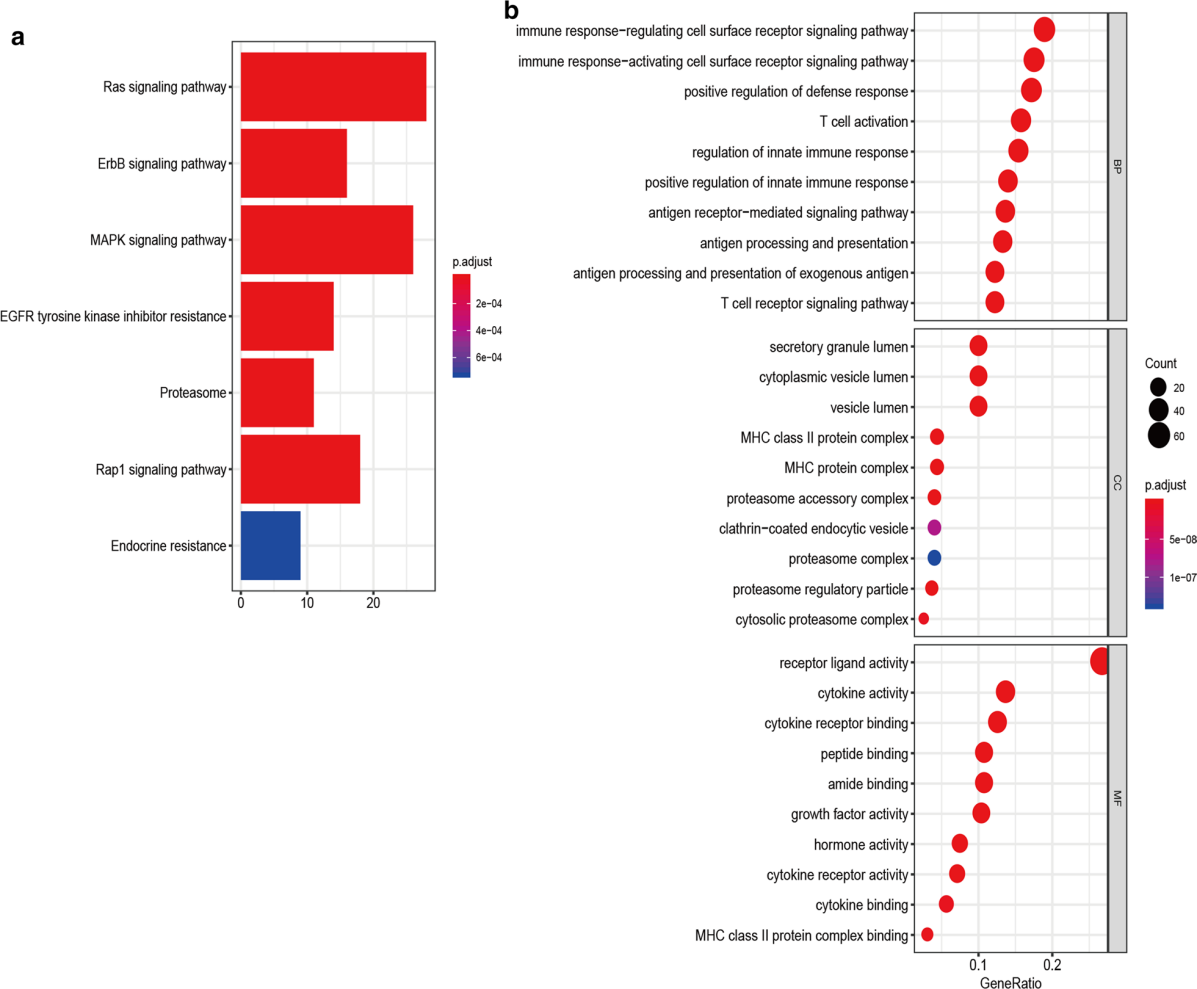
Functional enrichment analysis of immune-related genes in LUAD. **a** Kyoto Encyclopedia of Genes and Genomes (KEGG) pathways. **b** Gene ontology (GO) analysis on biological process (BP), cellular component (CC), and molecular function (MF)

299 survival-related immune genes were performed for gene enrichment analysis. As shown in Fig. 1, KEGG analysis demonstrated that MAPK, Ras, Rap1, ErbB pathways, EGFR tyrosine kinase inhibitor resistance, proteasome, and endocrine resistance were associated with these immune genes (Fig. 1a). GO analysis showed that the immune-related mechanisms such as immune response-activating/regulating cell surface receptor signaling pathway, T cell activation, T cell receptor signaling pathway, antigen receptor-mediated signaling pathway, antigen processing and presentation, cytokine activity, and MHC protein complex etc. were enriched (Fig. 1b).

### Construction of a 10 immune-related gene signature

We finally applied the multivariate Cox regression model to select the final genes in the training cohort. Eventually, 10 immune-related genes were used to construct a prognostic model in LUAD. A prognostic index was established based on the expression level and the corresponding regression coefficient of each immune-related gene, the following formula was present: [FURIN* 0.1089] + [PSMD14*(− 0.2344)], [ARRB1*(− 0.2738)] + (TUBB3*0.1028) + (ADM*0.1159) + [ZAP70*(− 0.2977)] + [RFXAP*(− 0.2549)] + [SHC3*(− 0.1522)] +[BMP5*(− 0.0763)] + (CD40LG*0.1625).

### A 10 immune-related gene signature predicts survival in LUAD

**Fig. 2:**
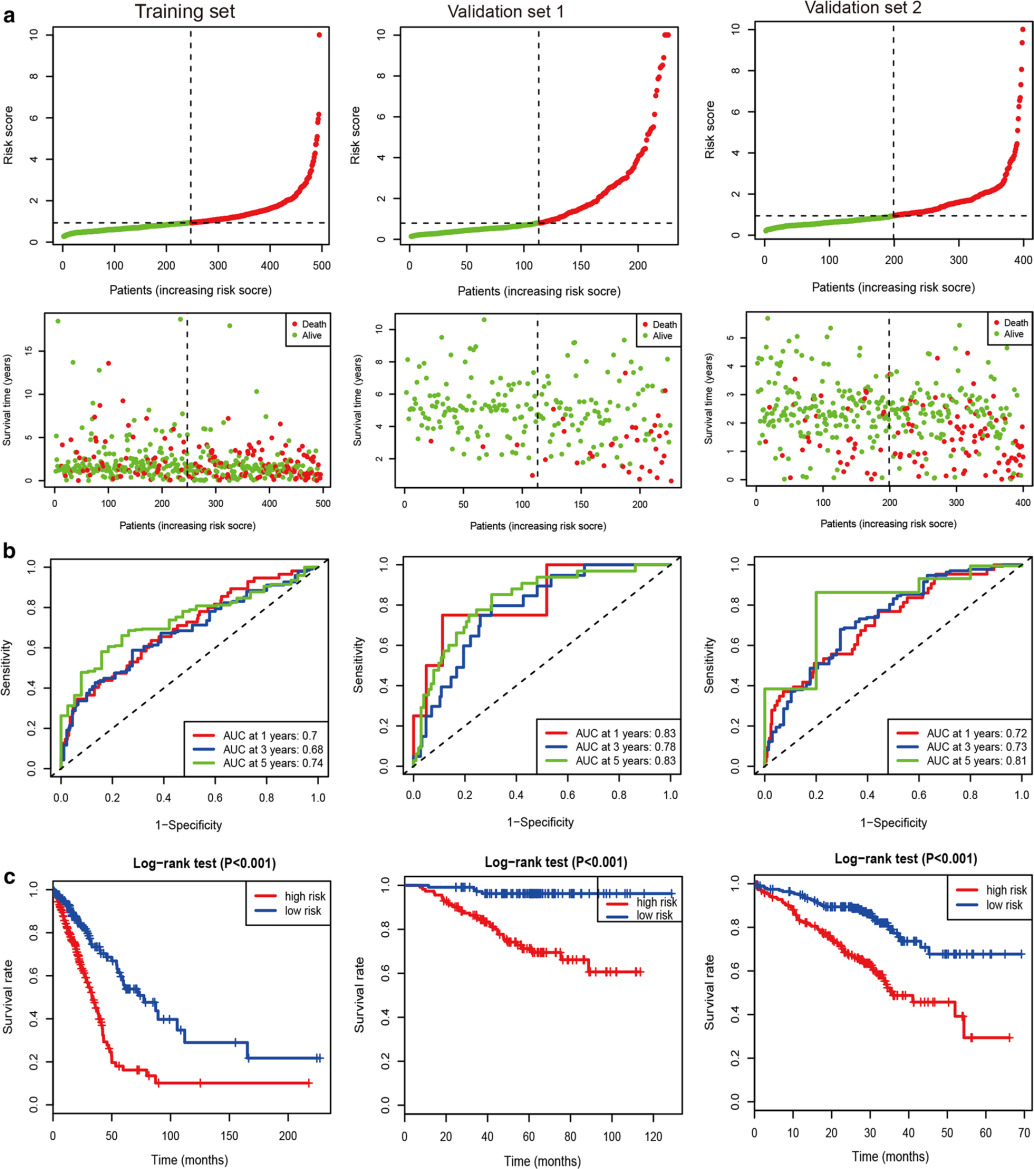
The prognostic signature based on 10 immune-related genes in LUAD. **a** The distribution and survival status of patients for the immune-related gene signature. **b** Time-dependent ROC results at 1, 3, and 5 years. **c** Kaplan–Meier curves between the high-risk and low-risk groups

The distribution and survival status of patients for the 10 immune-related gene signature were shown in the training and validation sets (Fig. 2a). Time-dependent ROC results were used to evaluate the predictive capacity of a 10 immune-related gene signature at 1, 3, and 5 years (Fig. 2b). The AUCs (Area under the ROC curve) for the long-time survival at 5 years were > 0.70 in the training set and the other two validation cohorts, indicating a good accuracy of the 10 immune-related gene signature for survival prediction. Kaplan–Meier curves showed that patients with LUAD in the high-risk group had closely shorter prognosis than patients with LUAD in the low-risk group (all *P* values < 0.001) (Fig. 2c).

### Independent prognostic and predictive value of the 10 immune-related gene signature

To examine whether our 10 immune-related gene signature was an independent molecular factor for survival prediction in the training and validation sets, univariate and multivariate Cox models were further carried out in this study (Table 2). Univariate Cox analysis showed that the high-risk group was closely associated with worse survival of LUAD in TCGA training set (494 cases: HR = 2.57, 95% CI 1.85–3.56, *P* < 0.001), validation set 1 (226 cases: HR = 9.11, 95% CI 3.21–25.82, *P* < 0.001), and validation set 2 (398 cases: HR = 2.80, 95% CI 1.87–4.17, *P* < 0.001). After adjusting for clinical and pathologic factors, further multivariate Cox analysis suggested that our immune signature was still a novel independent molecular indicator for predicting worse survival in LUAD in TCGA training set (HR = 1.68, 95% CI 1.11–2.54, *P* = 0.013), validation set 1 (HR = 8.63, 95% CI 2.95–25.21, *P* < 0.001), and validation set 2 (HR = 2.38, 95% CI 1.57–3.62, *P* < 0.001).

**Table 2:** Univariate and multivariate Cox analyses of the immune-related gene signature in one training set and two validation sets

Variables	Univariate Cox analysis		Multivariate Cox analysis	
	Hazard ratio (95% CI)	*P*	Hazard ratio (95% CI)	*P*
Training set				
Immune-related gene signature (high- vs. low-risk)	2.57 (1.85–3.56)	< 0.001	1.68 (1.11–2.54)	0.013
Age (≥ 65 vs. <65 years)	1.21 (0.88–1.66)	0.235		
Gender (male vs. female)	1.05 (0.77–1.43)	0.773		
Tumor stage (stage 3–4 vs. 1–2)	2.81 (2.03–3.88)	< 0.001	1.69 (1–2.86)	0.051
T (T 3–4 vs. 1–2)	2.41 (1.61–3.60)	< 0.001	1.48 (0.9–2.44)	0.123
M (positive vs. negative)	1.86 (1.06–3.25)	0.03	1.05 (0.53–2.06)	0.896
N (positive vs. negative)	2.64 (1.93–3.62)	< 0.001	1.71 (1.09–2.66)	0.019
Smoking (yes vs. no)	0.89 (0.57–1.41)	0.627		
Validation set 1				
Immune-related gene signature (high- vs. low-risk)	9.11 (3.21–25.82)	< 0.001	8.63 (2.95–25.21)	< 0.001
Age (≥ 65 vs. <65 years)	1.89 (0.96–3.71)	0.066		
Gender (male vs. female)	1.52 (0.78–2.96)	0.219		
Smoking (yes vs. no)	1.64 (0.84–3.20)	0.15		
KRAS mutation (yes vs. no)	0.87 (0.27–2.85)	0.817		
EGFR mutation (yes vs. no)	0.47 (0.24–0.93)	0.03	0.86 (0.43–1.73)	0.673
Validation set 2				
Immune-related gene signature (high- vs. low-risk)	2.80 (1.87–4.17)	< 0.001	2.38 (1.57–3.62)	< 0.001
Age (≥ 65 vs. <65 years)	1.38 (0.89–2.14)	0.151		
Gender (male vs. female)	1.55 (1.07–2.25)	0.02	1.42 (0.97–2.09)	0.075
Smoking (yes vs. no)	1.37 (0.60–3.14)	0.459		
Tumor stage (stage 3–4 vs. 1–2)	2.61 (1.74–3.91)	< 0.001	2.63 (1.74–3.97)	< 0.001
KRAS mutation (yes vs. no)	1.46 (1.00–2.12)	0.049	1.1 (0.74–1.62)	0.643
EGFR mutation (yes vs. no)	0.26 (0.10–0.71)	0.008	0.4 (0.14–1.10)	0.077
TP53 mutation (yes vs. no)	1.24 (0.82–1.86)	0.313		

*CI* confidence interval

### Predictive role of the 10 immune-related gene signature with the survival in various clinical and mutational characteristics

**Fig. 3:**
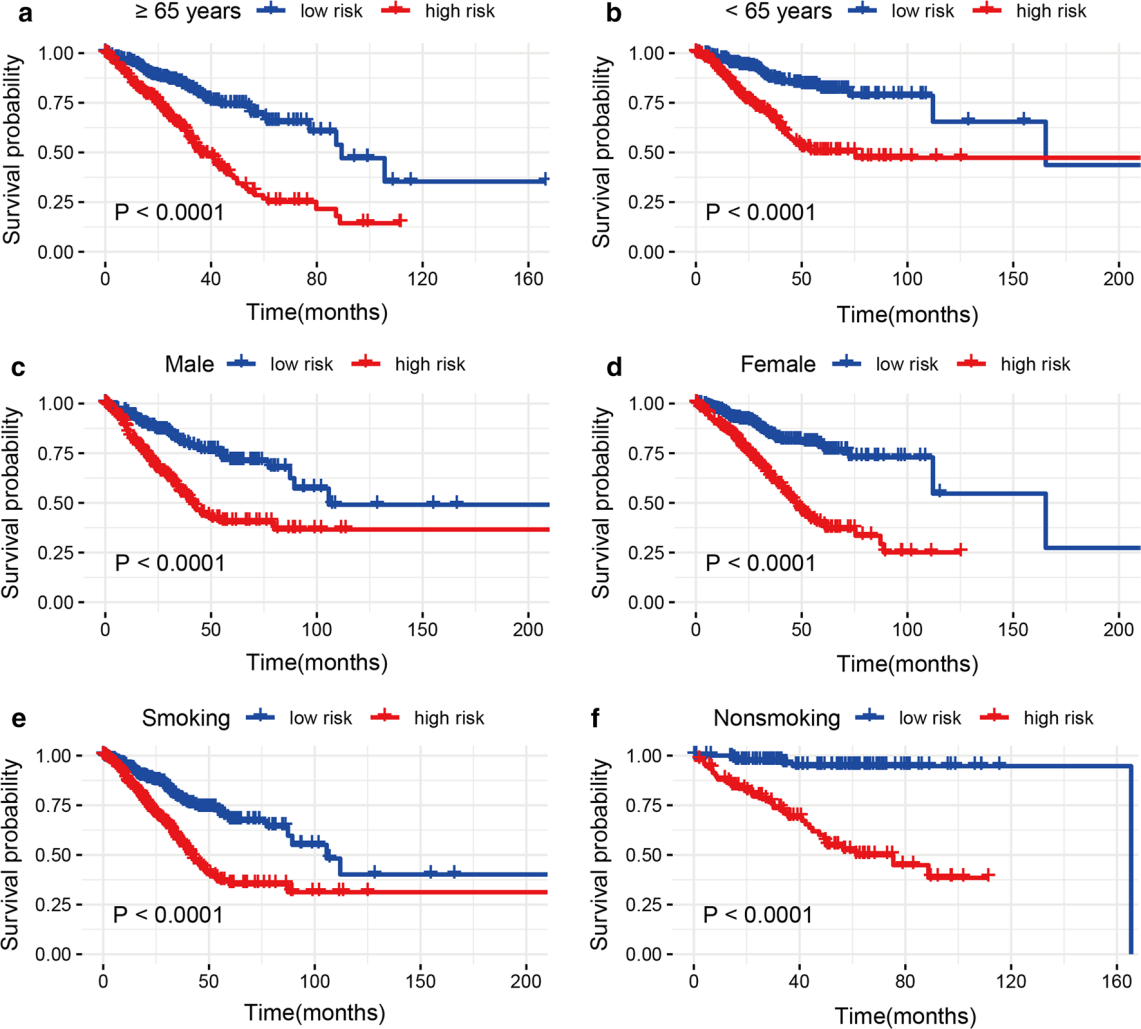
Kaplan-Meier analyses of LUAD patients with age, gender, and smoking behavior, including **a** ≥ 65 years, **b** < 65 years, **c** male, **d** female, **e** smoking, and **f** nonsmoking

**Fig. 4:**
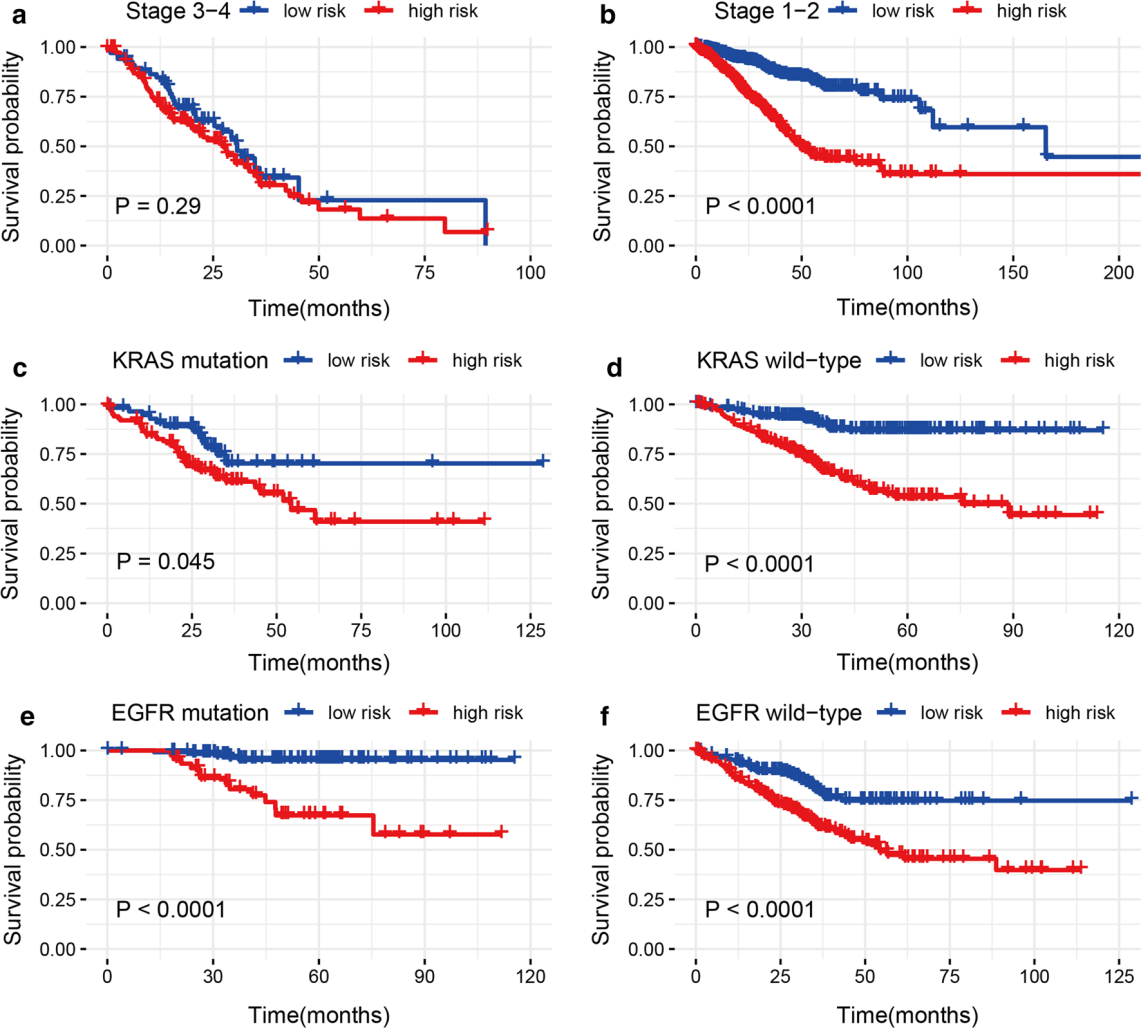
Kaplan–Meier analyses of LUAD patients with stage, KRAS mutation status, and EGFR mutation status, including **a** stage 3–4, **b** stage 1–2, **c** KRAS mutation, **d** KRAS wild-type, **e** EGFR mutation, and **f** EGFR wild-type

Stratification analyses were conducted based on age (≥ 65 vs. < 65 years), gender (male and female), smoking behavior (smoking and nonsmoking), tumor stage (stage 3–4: advanced-stage and stage 1–2: early-stage), KRAS mutation status (mutation and wild-type) and EGFR mutation status (mutation and wild-type) in the entire set (Figs. 3, 4). The cut-off value was 0.9325 and cases were divided into high- and low-risk groups. Because patients with various T, M, N, and TP53 mutation status were conducted in only a cohort, these clinical variables such as T, M, N, and TP53 mutation status were removed from subgroup analyses. For early-stage patients, the high-risk group indicated closely poor prognosis than the low-risk group (*P* < 0.05), but no significant prognostic relationship was observed between the high- and low-risk groups for advanced-stage patients (Fig. 4), which might cause owing to the small study population of advanced-stage patients. The results demonstrated that high-risk LUAD patients in each stratum of age, gender, smoking behavior, KRAS mutation, and EGFR mutation status presented worse survival than low-risk LUAD patients (all *P* values < 0.05) (Figs. 3, 4), suggesting that our 10 immune-related gene signature-based risk group stratification was still an effective tool for survival prediction in older or younger, male or female, and smoking or nonsmoking patients with LUAD, patients with KRAS mutation or KRAS wild-type, and patients with EGFR mutation or EGFR wild-type.

A stratification analysis of stage 1 and stage 2 tumors was also performed. The result showed that the high-risk group was closely correlated with poor prognosis in stage 1 and stage 2 LUAD tumors (*P* < 0.05) (Additional file 6: Figure S2), suggesting that our immune signature-based risk group stratification was still an effective tool for prognosis prediction in stage 1 and stage 2 tumors. Moreover, multivariate Cox analysis within early stages of LUAD tumors demonstrated that our immune signature was still an independent molecular factor for predicting survival (HR = 3.93, 95% CI 2.38–6.5, *P* < 0.0001) (Additional file 7: Table S5).

### Tumor-infiltrating immune cells

**Fig. 5:**
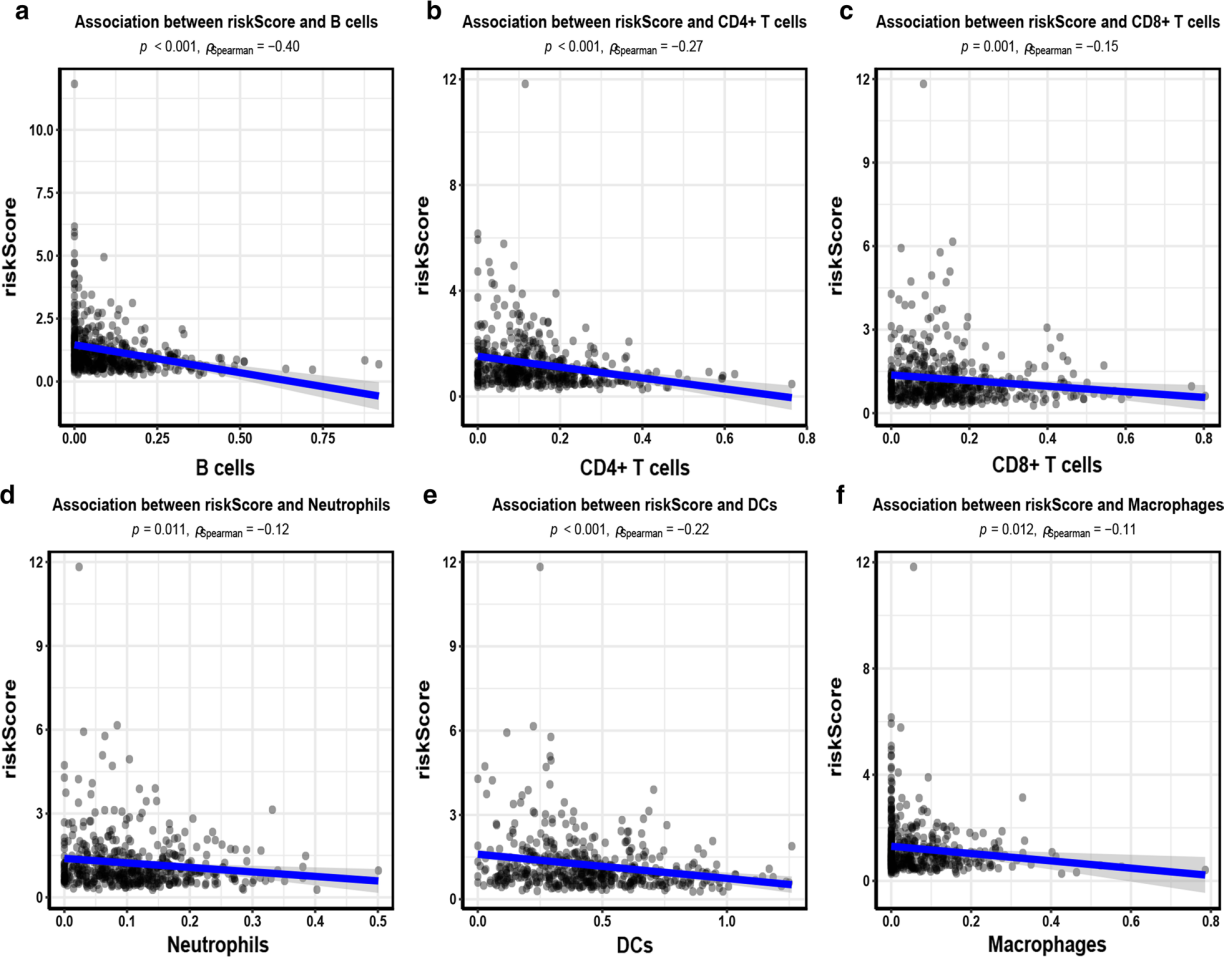
Correlation between our signature and tumor-infiltrating immune cells. **a** Association between risk score and B cells. **b** Association between risk score and CD4+ T cells. **c** Association between risk score and CD8+ T cells. **d** Association between risk score and Neutrophils. **e** Association between risk score and dendritic cells (DCs). **f** Association between risk score and Macrophages

We analyzed whether our immune-related gene signature was related to immune infiltration in LUAD, such as B cell, CD4 T cell, CD8 T cell, neutrophil, macrophage, and dendritic cell (DC). As shown in Fig. 5, our immune-related gene signature was negatively correlated with B cells (r = − 0.40, *P *< 0.001), CD4+ T cells (r = − 0.27, *P *< 0.001), DCs (r = − 0.22, *P *< 0.001), CD8+ T cells (r = − 0.15, *P *= 0.001), neutrophils (r = − 0.12, *P *= 0.011), and macrophages (r = − 0.11, *P *= 0.012).

### Association between our signature and immune checkpoint molecules

**Fig. 6:**
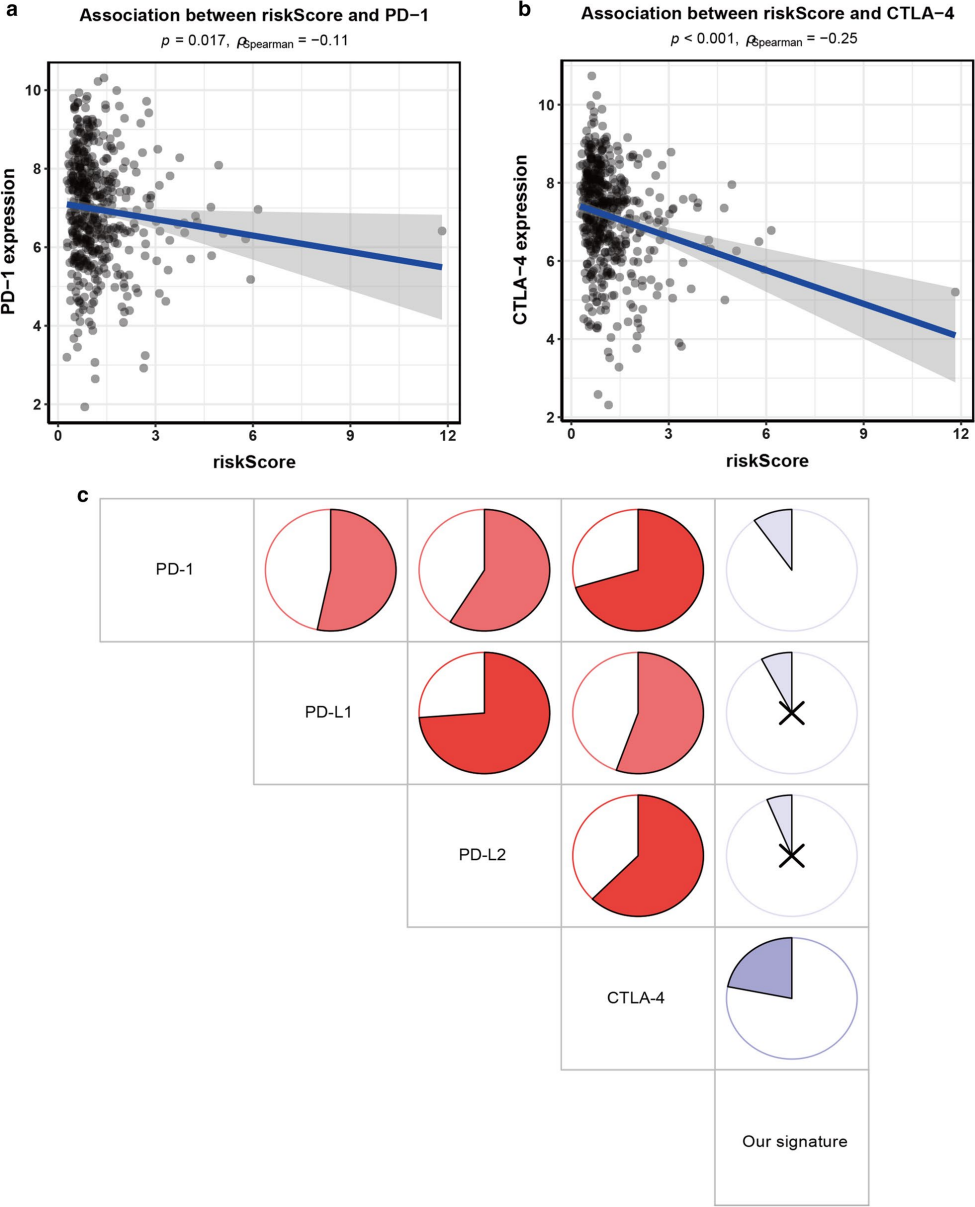
Correlation between our signature and known immune checkpoint genes. **a** Association between risk score and PD-1. **b** Association between risk score and CTLA-4. **c** Association of each immune checkpoint gene. “x” for no correlation; blue for negative correlation; red for positive correlation

Immune checkpoint blockade (ICB) therapy such as immune-checkpoint proteins programmed cell death 1 (PD-1), programmed cell death-ligand 1 (PD-L1), and cytotoxic T-lymphocyte-associated protein 4 (CTLA-4) targets has been reported to provide clinical benefits for cancer immunotherapy in some advanced-cancers such as melanoma and NSCLC [21, 22]. We investigated the correlation between our signature and the immune checkpoint molecules PD-1, PD-L1, PD-L2, and CTLA-4 in LUAD. The results showed that our signature was negatively associated with PD-1 (r = − 0.11, *P *= 0.017) and CTLA-4 (r = − 0.25, *P *< 0.001) (Fig. 6a, b). In addition, PD-1, PD-L1, PD-L2, and CTLA-4 were found to be coexpressed in LUAD (*P *< 0.001) (Fig. 6C).

## Discussion

The most frequent histological subtypes of lung cancer are LUAD and frequently occurs in females and nonsmoking people [23]. LUAD is related to distinct oncogene alterations. The common somatic gene aberrations are EGFR and KRAS mutations, and ALK rearrangements, which have been extensively reported and studied in LUAD [24, 25]. The frequency of oncogenic driver aberrations is different based on ethnicity, gender, and smoking behavior, which may result in differences in treatment efficacy [26]. In addition, in recent years reliable prognostic biomarkers have already appeared that are modifying the prognosis, which are applied to select groups of patients who are at high-risk and who may benefit from the personalized therapy [27–29], but their accuracy of survival estimation is not shown and remains limited. Therefore, it is imperative to identify precise biomarkers of LUAD and to select the appropriate immunotherapy for improving survival of this disease. In our current work, we constructed and validated a new prognostic signature based on 10 immune-related genes for survival prediction in patients with LUAD.

The immune system has a key role in cancer development progression [7, 8]. Such as, MAPK signaling involves in cell proliferation, apoptosis and immune escape, which contributes to the progression of the tumor [30]. Cytokines regulate tumor growth, survival, and invasion, and metastatic colonization [31]. LUAD shows altered T cell and NK cell compartments [32]. Choi et al. reported that high ImmuneScore was associated with favorable prognosis in LUAD [33]. However, immune-related molecular mechanisms involved in LUAD remain largely unclear. In this study, immune-related genes were found to be associated with immune regulation (i.e. immune response-activating/regulating cell surface receptor signaling pathway, T cell activation, and cytokine activity etc.) and biological pathways such as MAPK signaling in LUAD. Further 10 immune-related gene signature for survival prediction was identified in LUAD. Immune signatures can predict prognosis across solid tumors [34]. For example, a proposed clinical-immune signature is significantly correlated with worse prognosis and can be a promising biomarker for predicting overall survival in ovarian cancer [35]. The seven immune-related gene signature is significantly related to poor survival and can serve as a potential marker for reflecting the prognosis in clear renal clear cell carcinoma [36]. A prognostic immune signature can predict cervical cancer patients’ survival [37]. The nine immune-related gene signature is correlated with worse prognosis and is a potential predictive biomarker in hepatocellular carcinoma [38]. The previous findings were consistent with our current result that the high-risk patients were related to short survival time and worse survival of LUAD. Then, multivariate Cox analysis indicated that our immune signature remained an independent molecular indicator for survival prediction. These analyses confirmed that our immune signature was an effective and good performance for predicting prognosis in LUAD, which was reasonable and reliable.

This model for LUAD contained 10 immune-related genes. Among them, FURIN correlates with many cancer-related processes such as cell proliferation, migration, invasion, and angiogenesis, which promotes tumor progression [39]. FURIN has also a crucial function in the adaptive immunity [40]. PSMD14 induces cell cycle and senescence and may participate in the role of the proteasome [41, 42]. ARRB1 mediates signaling pathways and correlates with the regulation of DNA damage response [43]. ARRB1 participates in cell invasion and proliferation, thereby contributing to NSCLC progression [44]. TUBB3 is a key mechanism of drug resistance and TUBB3 expression shows predictive value for the prognosis in many cancers [45]. SHC3 regulates signal transduction, thereby stimulating hepatocellular carcinoma proliferation, migration, invasion, and epithelial-to-mesenchymal transition (EMT) [46]. BMP5 activates multiple signaling pathways such as p38 MAPK signaling [47]. CD40LG polymorphism is related to various immunological disorders such as tumors [48]. Adrenomedullin (ADM) correlates with cellular growth, anti-apoptotic property, angiogenesis, tumor cell motility and metastasis, inflammatory and immune responses. And ADM is also a significant factor for worse survival in some cancers [49, 50]. ZAP70 is related to the T cell antigen receptor (TCR) complex and is required for T cell activation [51, 52]. RFXAP is a key transcription factor for major histocompatibility complex (MHC) II [53]. On the basis of the above findings, our work integrated 10 immune-related genes into a single panel, found patients in the high immune-risk group associated with worse survival, and confirmed the predictive value of our signature in LUAD. The high abundance of T cell and B-cell is correlated with improved survival in many cancer types, including lung cancer [54]. A high level of tumor-infiltrating lymphocytes is associated with better prognosis of LUAD [55]. High B-cell and CD8+ T cell infiltration is reported to be associated with favorable prognosis in LUAD [54, 56]. The immune checkpoint molecules PD-1 and CTLA-4 treatment is a promising cancer immunotherapy approach for clinical benefits [21]. Studies have reported the coexpression PD-1, PD-L1, PD-L2, and CTLA-4 immune checkpoint molecules [57]. In our study, we also found PD-1, PD-L1, PD-L2, and CTLA-4 immune checkpoint molecules were coexpressed in LUAD. Although our signature was negatively correlated with tumor-infiltrating immune cells and PD-1 and CTLA-4, the correlation was not very significant. Biomarkers of response to ICB do not frequently only focus on the target molecules, but rather inflammatory/interferon signaling cascades [58–60]. Thus, our signature may not have a significant response to immune checkpoint molecules. Additional data on the association of our model with other signatures (either a cytolytic signature, IFNG signature, chemokine signature, or the tumor inflammation signature) could be needed in the future.

The common somatic mutations are EGFR and KRAS in LUAD [24]. Hsiao et al. reported that EGFR mutations were closely correlated with therapeutic efficacy and progression-free survival in LUAD [61]. We further evaluated the predictive effect of our 10 immune-related gene signature in different clinical and molecular features. We found that our 10 immune-related gene signature remained a strongly powerful tool for predicting prognosis in older or younger, male or female, and smoking or nonsmoking patients with LUAD, patients with KRAS mutation or KRAS wild-type, and patients with EGFR mutation or EGFR wild-type. Our immune signature in the high-risk group was observed to be associated with worse prognosis for early-stage tumors. Further multivariate Cox analysis showed that our immune signature was still an independent molecular indicator for survival prediction in early-stage LUAD tumors.

These are several limitations in this work that should be noted. First, our study was a retrospective design, more prospective clinical data sets as possible are needed to validate our result in the future. Second, our immune signature was developed by numerous genes, further biological functions are warranted to be further explored in LUAD. Third, our immune signature was calculated based on the gene expression values. Thus, intra-tumor heterogeneity supported by genetic and phenomenological data, which could cause sampling bias.

## Conclusion

In conclusion, a 10 immune-related gene signature was built for prognosis prediction in LUAD. This study demonstrated that this 10 immune-related gene signature may become a promising prognostic marker for LUAD, especially in early-stage patients, which could facilitate the personalized therapy and serve new immunotherapy of LUAD. Further studies are still required to prove this signature in LUAD in the future.

## Supplementary information

**Additional file 1: Table S1.** 299 survival-related genes for enrichment analysis.

**Additional file 2: Table S2.** Immune-related genes from the ImmPort database.

**Additional file 3: Table S3.** 52 potential survival-related immune genes from three cohorts.

**Additional file 4: Figure S1.** Venn diagram of the overlapping immune-related genes with the survival using univariate Cox analysis with P < 0.05 from three data sets.

**Additional file 5: Table S4.** Final 10 immune-related genes in the current model.

**Additional file 6: Figure S2.** Kaplan–Meier analyses of our immune signature in stage 1 and stage 2 tumors, including (A) stage 1 lung adenocarcinoma (LUAD) and (B) stage 2 LUAD.

**Additional file 7: Table S5.** Multivariate Cox analyses of our immune signature in early-stage lung adenocarcinoma (LUAD).

## Data Availability

The data sets used are available from the corresponding author on a reasonable request.

## Abbreviations

LUAD: Lung adenocarcinoma
NSCLC: Non-small cell lung cancer
LUSC: Lung squamous cell carcinoma
EGFR: Epidermal growth factor receptor
TCGA: The Cancer Genome Atlas
TMM: Trimmed Mean of M-values
GEO: The Gene Expression Omnibus
OS: Overall survival
ImmPort: The Immunology Database and Analysis Portal
GO: Gene ontology
KEGG: The Kyoto Encyclopedia of Genes and Genomes
ROC: Receiver-operating characteristic
ADM: Adrenomedullin
EMT: Epithelial-to-mesenchymal transition
MHC: Major histocompatibility complex
DC: Dendritic cell
TME: Tumor microenvironment
ICB: Immune checkpoint blockade
PD-1: Programmed cell death 1
PD-L1: Programmed cell death-ligand 1
PD-L2: Programmed cell death-ligand 2
CTLA-4: Cytotoxic T-lymphocyte-associated protein 4
