# Supplementary material for: A new immune signature for survival prediction and immune checkpoint molecules in lung adenocarcinoma
Source: J Transl Med. 2020 Mar 6;18:118. doi: 10.1186/s12967-020-02286-z (PMC7060601; doi:10.1186/s12967-020-02286-z)
Supplement: Supplementary file 1 — Additional file 1: Table S1. 299 survival-related genes for enrichment analysis. [file 12967_2020_2286_MOESM1_ESM.docx]

**Table S1. 299 survival-related genes for enrichment analysis.**

**Gene symbol**

DKK1

EPGN

VEGFC

CXCL17

VIPR1

SHC1

BMP5

ADIPOR2

ANGPTL4

WFDC2

RAET1L

ADM

FCGRT

GHRL

ARRB1

SCGB3A1

FGF5

GIP

NRAS

NR0B2

RFXAP

BIRC5

INSL4

JAG1

AGER

S100A16

ADRB2

CD1C

PSMD2

UMODL1

SHC3

SFTPD

NEDD4

FURIN

CACYBP

STC1

HLA-DMA

PSMD11

MAP3K8

TNFRSF13B

CRHR2

HSPA4

LGR4

CD1D

SEMA3C

NR3C2

PGC

IFNE

SEMA4A

CD1E

KRAS

CD19

CD1B

IL11RA

TMEM173

CX3CR1

LIFR

CMTM7

IL24

CR2

XCR1

PAK2

INHA

GDF15

IL1R2

IL22RA1

PLSCR1

NMB

RAET1E

KL

GPI

GMFB

CIITA

CD40LG

PTGDS

TUBB3

CTF1

TLR2

GAL

RFX5

PTPN6

PSMC6

SERPIND1

LCN10

IL1A

IL20RB

IL16

LCN15

S100A10

EREG

NTS

RAC1

F2RL1

CD79B

TRAV34

UCN2

PSMD1

CSF2

CAMP

OAS1

NOD1

BTK

CHIT1

ITGAL

S100P

SEMA4B

PRKCB

ACTG1

EPOR

CAT

PLCG1

GRAP2

LEFTY2

CCL20

TAP2

CD22

PSME3

SFTPA2

OXTR

HLA-DOB

PSMD7

PIK3R3

CTSG

HLA-DMB

CSF3R

ADRB1

NFKBIZ

HNF4G

CGB8

IL33

RARG

PTHLH

CMA1

SORT1

SEMA3A

ITGAV

PSMC5

NOX4

TSLP

HTR3C

PLCG2

HLA-DQA1

IL3RA

GREM1

TNFRSF10C

INHBA

NCR3

GPR17

PIK3CD

KITLG

LCNL1

TNFRSF13C

PSMC1

PIK3CA

EDN3

PLAUR

IL12B

DMBT1

SFTPA1

TNFSF13

BMP1

CRP

TPM2

CD79A

SYTL1

RBP5

PPIA

RXFP1

RORC

LCN1

TRBV14

NDRG1

IL11

TRAV21

TRBV12-5

HLA-DPB1

TNFSF12

PAK4

S100A7

PLXNA2

NR2F2

SEMA6C

FGA

TRBV2

CCR7

TGFBR1

TRBV10-2

LAT

PSMC4

FLT3

VGF

CMTM5

NGF

LIMS1

SP1

TRAV8-6

CCL17

PTPN11

CX3CL1

KIR2DL1

TRAV14DV4

STC2

ZAP70

IAPP

GAST

NFYB

HSP90AA1

HLA-DRB5

BMPR1B

FGF12

CD74

NFATC1

HLA-DOA

RELB

TRBV18

RAET1G

HLA-DRA

HLA-DQB1

TRIM22

TBK1

TRBV3-1

CCR6

TAP1

LTB4R2

GMFG

HRG

LTBR

RXRB

KIR3DL2

LTB4R

NLRX1

STAT1

CCR4

TNFRSF19

IGKV2D-30

RXRG

TRBC1

LTB

IKBKB

CCL14

MIF

SCG2

AP3B1

FGFR2

HLA-DRB1

RELA

HSPA2

CD1A

ROBO2

RXFP2

HDGF

PTGDR

GLP2R

THRA

PTK2B

MASP2

MC1R

PSMD14

IFI30

TRBV7-4

PRTN3

KCNH2

S100A11

HGF

IL17RB

FGR

TMSB15B

MAPT

TNFRSF1A

AGTR2

NCK1

IL5RA

KLRC3

IL2

ANGPTL6

SEMA4G

GSK3B

GDF5

TRBV13

TRBV7-6

PAK1

HLA-DPA1

QRFP

IGKV1-8

RETN

GDF10

TNFSF11

NOX5

PI15

TCHHL1

IGHD2-2

TMSB10

FGF2

LANCL1

RORA

PSMD8

BLNK

LTBP1

TRAV8-2
