# Supplementary material for: A new immune signature for survival prediction and immune checkpoint molecules in lung adenocarcinoma
Source: J Transl Med. 2020 Mar 6;18:118. doi: 10.1186/s12967-020-02286-z (PMC7060601; doi:10.1186/s12967-020-02286-z)
Supplement: Supplementary file 2 — Additional file 2: Table S2. Immune-related genes from the ImmPort database. [file 12967_2020_2286_MOESM2_ESM.docx]

**Table S2. Immune-related genes from the ImmPort database.**

| **Gene symbol** |
| --- |
| AZGP1 |
| B2M |
| CALR |
| CANX |
| CD1A |
| CD1B |
| CD1C |
| CD1D |
| CD1E |
| CD4 |
| CD8A |
| CD8B |
| CD74 |
| CREB1 |
| CTSB |
| CTSE |
| CTSL1 |
| CTSS |
| FCER1G |
| FCGRT |
| PDIA3 |
| HFE |
| HLA-A |
| HLA-B |
| HLA-C |
| HLA-DMA |
| HLA-DMB |
| HLA-DOA |
| HLA-DOB |
| HLA-DPA1 |
| HLA-DPB1 |
| HLA-DQA1 |
| HLA-DQA2 |
| HLA-DQB1 |
| HLA-DRA |
| HLA-DRB1 |
| HLA-DRB3 |
| HLA-DRB4 |
| HLA-DRB5 |
| HLA-E |
| HLA-F |
| HLA-G |
| HLA-H |
| MR1 |
| HSPA1A |
| HSPA1B |
| HSPA1L |
| HSPA2 |
| HSPA4 |
| HSPA5 |
| HSPA6 |
| HSPA8 |
| HSP90AA1 |
| HSP90AB1 |
| ICAM1 |
| IFNA1 |
| IFNA2 |
| IFNA4 |
| IFNA5 |
| IFNA6 |
| IFNA7 |
| IFNA8 |
| IFNA10 |
| IFNA13 |
| IFNA14 |
| IFNA16 |
| IFNA17 |
| IFNA21 |
| IFNG |
| KIR2DL1 |
| KIR2DL2 |
| KIR2DL3 |
| KIR2DL4 |
| KIR2DS1 |
| KIR2DS3 |
| KIR2DS4 |
| KIR2DS5 |
| KIR3DL1 |
| KIR3DL2 |
| KLRC1 |
| KLRC2 |
| KLRC3 |
| KLRD1 |
| LTA |
| CIITA |
| MICA |
| MICB |
| NFYA |
| NFYB |
| NFYC |
| LGMN |
| PSMB8 |
| PSMC1 |
| PSMC2 |
| PSMC3 |
| PSMC4 |
| PSMC5 |
| PSMC6 |
| PSMD1 |
| PSMD2 |
| PSMD3 |
| PSMD4 |
| PSMD5 |
| PSMD7 |
| PSMD8 |
| PSMD10 |
| PSMD11 |
| PSMD13 |
| PSME1 |
| PSME2 |
| RELB |
| RFX5 |
| RFXAP |
| SLC10A2 |
| TAP1 |
| TAP2 |
| TAPBP |
| THBS1 |
| SHFM1 |
| KLRC4 |
| AP3B1 |
| RFXANK |
| PSMD6 |
| PSME3 |
| PSMD14 |
| CLEC4M |
| IFI30 |
| PROCR |
| ADRM1 |
| KIAA0368 |
| TRPC4AP |
| CD209 |
| UBXN1 |
| ERAP1 |
| TAPBPL |
| KIR2DL5A |
| ERAP2 |
| ULBP3 |
| ULBP2 |
| ULBP1 |
| KIR3DL3 |
| RAET1E |
| RAET1L |
| UBR1 |
| RAET1G |
| PDIA2 |
| HAMP |
| PI3 |
| CAMP |
| DEFB4 |
| PPBP |
| REG3G |
| CXCL14 |
| CXCL16 |
| SLPI |
| IL8 |
| CXCL10 |
| CXCL9 |
| CXCL5 |
| CXCL11 |
| CXCL6 |
| CXCL1 |
| CXCL12 |
| CXCL13 |
| CXCL2 |
| PF4 |
| XCL1 |
| CXCL3 |
| DEFB103A |
| CCL13 |
| CCL1 |
| DEFB1 |
| CCL8 |
| ELANE |
| DEFB103B |
| DEFA3 |
| DEFA1 |
| TMSB10 |
| DEFA6 |
| DEFA5 |
| DEFA4 |
| LCN2 |
| LCN1 |
| COLEC10 |
| BPI |
| S100A9 |
| S100A8 |
| DCD |
| LCN6 |
| S100A12 |
| HTN3 |
| LCN8 |
| LOC728358 |
| CCR10 |
| CELA1 |
| DEFB106A |
| PENK |
| BPIL2 |
| MMP12 |
| BPIL3 |
| LEAP2 |
| SFTPD |
| LCN9 |
| BPIL1 |
| PTGDS |
| TMSB4X |
| PGLYRP1 |
| ZC3HAV1 |
| TMSB15A |
| S100B |
| S100A13 |
| S100A6 |
| DEFB119 |
| DEFB107A |
| DEFB105A |
| SERPIND1 |
| DEFB129 |
| DEFB127 |
| S100P |
| S100A7 |
| DEFB104A |
| DEFB126 |
| DEFB106B |
| DEFB104B |
| DEFB107B |
| PGLYRP3 |
| PGLYRP2 |
| S100A10 |
| S100A2 |
| DEFB125 |
| DEFB123 |
| DEFB105B |
| DEFB132 |
| C20orf185 |
| LCN12 |
| PGLYRP4 |
| S100A11 |
| S100A5 |
| S100A3 |
| S100A1 |
| DEFB128 |
| DEFB108B |
| HTN1 |
| LMBR1L |
| S100A7A |
| DEFB118 |
| COLEC12 |
| TMSB4Y |
| DEFB131 |
| DEFB134 |
| DEFB130 |
| DEFB124 |
| DEFB121 |
| DEFB116 |
| DEFB115 |
| DEFB114 |
| DEFB113 |
| DEFB112 |
| DEFB110 |
| TMSB15B |
| DEFB133 |
| S100Z |
| MAVS |
| TMSL3 |
| S100A14 |
| LCN10 |
| S100A16 |
| DEFB137 |
| DEFB136 |
| DEFB117 |
| DEFB111 |
| ZC3HAV1L |
| S100A7L2 |
| LOC731414 |
| LOC730963 |
| COLEC2 |
| DEFB4P |
| C20orf186 |
| IFNAR1 |
| AZU1 |
| LOC729523 |
| LOC100130154 |
| LOC100134379 |
| LOC100134289 |
| LOC100129216 |
| DEFA1A3 |
| LOC100131433 |
| LCN1L1 |
| S100G |
| LOC648637 |
| LOC100130969 |
| LOC100133267 |
| LOC100133128 |
| LOC100128174 |
| TCHHL1 |
| TINAGL1 |
| IFNGR1 |
| SLC22A17 |
| WFIKKN1 |
| WFDC2 |
| IL6 |
| UMODL1 |
| TGFB1 |
| PF4V1 |
| MMP9 |
| KAL1 |
| TLR4 |
| SPAG11B |
| A2M |
| NFKB1 |
| APOBEC3G |
| FABP6 |
| NOD2 |
| MBL2 |
| SFTPA1B |
| RBP1 |
| TLR2 |
| SLC40A1 |
| PLAU |
| IL1B |
| PAEP |
| HFE2 |
| MUC5AC |
| OBP2A |
| PLTP |
| MX1 |
| DDX58 |
| IL29 |
| IRF3 |
| SFTPA2 |
| SFTPA2B |
| LPA |
| LBP |
| RBP4 |
| SFTPA1 |
| NOX4 |
| LTF |
| IFNB1 |
| RBP5 |
| FABP7 |
| FABP5 |
| FABP3 |
| FABP2 |
| FABP4 |
| R3HDML |
| C20orf71 |
| C20orf114 |
| OASL |
| CRABP2 |
| CRABP1 |
| RBP7 |
| DUOX1 |
| OBP2B |
| RBP2 |
| LCN15 |
| CETP |
| FABP12 |
| FABP9 |
| PLUNC |
| LCNL1 |
| C8G |
| SPAG11A |
| PI15 |
| NOX1 |
| PMP2 |
| APOD |
| ORM2 |
| ORM1 |
| TNF |
| CTSG |
| PRTN3 |
| MAPK1 |
| PML |
| AEN |
| CYBB |
| C20orf70 |
| ISG20 |
| BCL3 |
| ISG20L2 |
| NOX5 |
| NOX3 |
| DUOX2 |
| TLR3 |
| TFRC |
| IFIH1 |
| LRP1 |
| TRIM5 |
| IDO1 |
| GDF15 |
| NEDD4 |
| ADIPOQ |
| STAT3 |
| STAT1 |
| IL28A |
| SOCS3 |
| SEMG1 |
| TNFSF10 |
| CCL20 |
| SOCS1 |
| RNASEL |
| IRF1 |
| IL15 |
| APOBEC3F |
| RARRES3 |
| CHIT1 |
| CD40 |
| TLR7 |
| PPIA |
| ZYX |
| NLRX1 |
| PGC |
| VEGFA |
| IKBKE |
| ISG15 |
| DHX58 |
| TNFAIP3 |
| TFR2 |
| FCN2 |
| MUC4 |
| F2R |
| ELN |
| IL27 |
| MAPT |
| LYZ |
| CCL5 |
| LEP |
| CYLD |
| KLKB1 |
| CST4 |
| CSRP1 |
| MAPK14 |
| JUN |
| ITGAV |
| IRF5 |
| CCR6 |
| IL12B |
| TLR8 |
| GNLY |
| CD81 |
| EIF2AK2 |
| APOM |
| CACYBP |
| NOD1 |
| MAPK8 |
| MAPK3 |
| BST2 |
| BPHL |
| PLA2G2A |
| GRN |
| NEWENTRY |
| PDGFRA |
| GNAI1 |
| WNT5A |
| FURIN |
| ADAR |
| TYK2 |
| NOS2 |
| TRAF3 |
| TPT1 |
| TPM2 |
| NEO1 |
| AHNAK |
| TLR1 |
| TK2 |
| PRDX2 |
| MX2 |
| FGF2 |
| FGA |
| TCF7L2 |
| F2RL1 |
| DAK |
| MSR1 |
| NFKBIZ |
| LMBR1 |
| SPINLW1 |
| SRC |
| MPO |
| ELAVL1 |
| ROBO3 |
| SP1 |
| SOD1 |
| PDF |
| DLL4 |
| ECD |
| SLC11A1 |
| DMBT1 |
| TMEM173 |
| SKIV2L |
| SEMG2 |
| DES |
| DCK |
| DAXX |
| TNFRSF10A |
| TNFRSF10B |
| EED |
| CCL4 |
| LIMS1 |
| LALBA |
| APOBEC3H |
| TMPRSS6 |
| SPINK5 |
| MARCO |
| BECN1 |
| TNFSF11 |
| KNG1 |
| CSK |
| KLRK1 |
| KCNH2 |
| JUND |
| JAK1 |
| CLDN4 |
| CCL28 |
| RNASE3 |
| RN7SL1 |
| IRF7 |
| IREB2 |
| ILK |
| IL18 |
| IL17A |
| LTB4R |
| APOBEC3A |
| MASP2 |
| TRIM27 |
| RELA |
| IL7R |
| IL1A |
| PTX3 |
| IFNAR2 |
| IFN1@ |
| SYTL1 |
| APOBEC3C |
| DDX17 |
| PTGS2 |
| HTR1A |
| 7-Sep |
| CD40LG |
| CD14 |
| MASP1 |
| PROC |
| MAP2K2 |
| MAP2K1 |
| HRG |
| NDRG1 |
| IRF9 |
| TRIM22 |
| LANCL1 |
| PPP4C |
| HMOX1 |
| HMGB1 |
| RNASE7 |
| ABCC4 |
| HGF |
| HDAC1 |
| IL28RA |
| PLSCR1 |
| BACH2 |
| TANK |
| PIK3CG |
| ARRB1 |
| RSAD2 |
| STAB2 |
| TBK1 |
| PDYN |
| PDGFRB |
| PDCD1 |
| PCSK2 |
| PCSK1 |
| ARG2 |
| AQP9 |
| FASLG |
| APOH |
| BIRC5 |
| ANXA6 |
| IL22 |
| VTN |
| VIM |
| VCAM1 |
| PRDX1 |
| GFAP |
| GBP2 |
| ALB |
| SLC29A3 |
| OAS1 |
| AGER |
| UNC93B1 |
| TNFSF4 |
| NOS1 |
| ACTG1 |
| ACTA1 |
| ACO1 |
| SERPINA3 |
| IL8RA |
| CCL15 |
| CCL14 |
| CCL16 |
| CCL19 |
| CCL18 |
| CCL17 |
| CCL26 |
| CCL22 |
| CCR3 |
| CCL4L2 |
| CCBP2 |
| CCR7 |
| CCL27 |
| CCR8 |
| CCRL1 |
| CCL2 |
| CCL21 |
| CCL7 |
| CCL3 |
| CCL11 |
| CCR5 |
| CCL23 |
| CCL25 |
| CCL3L3 |
| CCL4L1 |
| CCL3L1 |
| CCR1 |
| CCL24 |
| XCL2 |
| CXCR4 |
| CXCR6 |
| CCR4 |
| FAM19A5 |
| FAM19A3 |
| FAM19A4 |
| FAM19A1 |
| FAM19A2 |
| CCL14-CCL15 |
| PTK2B |
| IL4 |
| CDH1 |
| LTBP1 |
| IL13 |
| IL10 |
| IL2 |
| PPARG |
| FGR |
| MIF |
| CRP |
| JAK2 |
| PTK2 |
| PTGDR |
| CD86 |
| HCK |
| VDR |
| OLR1 |
| ADRBK1 |
| TXK |
| RNASE2 |
| CD79A |
| CD79B |
| LYN |
| SYK |
| BTK |
| BLNK |
| VAV3 |
| VAV1 |
| VAV2 |
| RAC1 |
| RAC2 |
| RAC3 |
| PPP3CA |
| PPP3CB |
| PPP3CC |
| CHP |
| PPP3R1 |
| PPP3R2 |
| CHP2 |
| NFAT5 |
| NFATC1 |
| NFATC2 |
| NFATC3 |
| NFATC4 |
| HRAS |
| KRAS |
| NRAS |
| FOS |
| CARD11 |
| BCL10 |
| MALT1 |
| CHUK |
| IKBKB |
| IKBKG |
| NFKBIA |
| NFKBIB |
| NFKBIE |
| CD19 |
| CR2 |
| PIK3R5 |
| PIK3R1 |
| PIK3R2 |
| PIK3R3 |
| PIK3CA |
| PIK3CB |
| PIK3CD |
| AKT3 |
| AKT1 |
| AKT2 |
| GSK3B |
| INPP5D |
| CD22 |
| CD72 |
| PTPN6 |
| LILRB3 |
| FCGR2B |
| RASGRP3 |
| PLCG2 |
| PRKCB |
| IFITM1 |
| IGH@ |
| IGHA1 |
| IGHA2 |
| IGHD |
| IGHD@ |
| IGHD1-1 |
| IGHD1-14 |
| IGHD1-20 |
| IGHD1-26 |
| IGHD1-7 |
| IGHD2-15 |
| IGHD2-2 |
| IGHD2-21 |
| IGHD2-8 |
| IGHD3-10 |
| IGHD3-16 |
| IGHD3-22 |
| IGHD3-3 |
| IGHD3-9 |
| IGHD4-11 |
| IGHD4-17 |
| IGHD4-23 |
| IGHD4-4 |
| IGHD5-12 |
| IGHD5-18 |
| IGHD5-24 |
| IGHD5-5 |
| IGHD6-13 |
| IGHD6-19 |
| IGHD6-25 |
| IGHD6-6 |
| IGHD7-27 |
| IGHE |
| IGHG1 |
| IGHG2 |
| IGHG3 |
| IGHG4 |
| IGHJ@ |
| IGHJ1 |
| IGHJ2 |
| IGHJ3 |
| IGHJ4 |
| IGHJ5 |
| IGHJ6 |
| IGHM |
| IGHV@ |
| IGHV1-18 |
| IGHV1-2 |
| IGHV1-24 |
| IGHV1-3 |
| IGHV1-45 |
| IGHV1-46 |
| IGHV1-58 |
| IGHV1-69 |
| IGHV1-8 |
| IGHV1-C |
| IGHV1-F |
| IGHV2-26 |
| IGHV2-5 |
| IGHV2-70 |
| IGHV3-11 |
| IGHV3-13 |
| IGHV3-15 |
| IGHV3-16 |
| IGHV3-20 |
| IGHV3-21 |
| IGHV3-23 |
| IGHV3-30 |
| IGHV3-30-3 |
| IGHV3-30-5 |
| IGHV3-33 |
| IGHV3-35 |
| IGHV3-38 |
| IGHV3-43 |
| IGHV3-48 |
| IGHV3-49 |
| IGHV3-53 |
| IGHV3-64 |
| IGHV3-66 |
| IGHV3-7 |
| IGHV3-72 |
| IGHV3-73 |
| IGHV3-74 |
| IGHV3-9 |
| IGHV3-D |
| IGHV3-H |
| IGHV4-28 |
| IGHV4-30-1 |
| IGHV4-30-2 |
| IGHV4-30-4 |
| IGHV4-31 |
| IGHV4-34 |
| IGHV4-39 |
| IGHV4-4 |
| IGHV4-59 |
| IGHV4-61 |
| IGHV4-B |
| IGHV5-51 |
| IGHV5-A |
| IGHV6-1 |
| IGHV7-4-1 |
| IGHV7-81 |
| IGK@ |
| IGKC |
| IGKDEL |
| IGKJ@ |
| IGKJ1 |
| IGKJ2 |
| IGKJ3 |
| IGKJ4 |
| IGKJ5 |
| IGKV@ |
| IGKV1-12 |
| IGKV1-13 |
| IGKV1-16 |
| IGKV1-17 |
| IGKV1-27 |
| IGKV1-33 |
| IGKV1-37 |
| IGKV1-39 |
| IGKV1-5 |
| IGKV1-6 |
| IGKV1-8 |
| IGKV1-9 |
| IGKV1D-12 |
| IGKV1D-13 |
| IGKV1D-16 |
| IGKV1D-17 |
| IGKV1D-33 |
| IGKV1D-37 |
| IGKV1D-39 |
| IGKV1D-42 |
| IGKV1D-43 |
| IGKV1D-8 |
| IGKV2-24 |
| IGKV2-28 |
| IGKV2-30 |
| IGKV2-40 |
| IGKV2D-24 |
| IGKV2D-28 |
| IGKV2D-29 |
| IGKV2D-30 |
| IGKV2D-40 |
| IGKV3-11 |
| IGKV3-15 |
| IGKV3-20 |
| IGKV3-7 |
| IGKV3D-11 |
| IGKV3D-15 |
| IGKV3D-20 |
| IGKV3D-7 |
| IGKV4-1 |
| IGKV5-2 |
| IGKV6-21 |
| IGKV6D-21 |
| IGKV6D-41 |
| IGL@ |
| IGLC@ |
| IGLC1 |
| IGLC2 |
| IGLC3 |
| IGLC6 |
| IGLC7 |
| IGLJ@ |
| IGLJ1 |
| IGLJ2 |
| IGLJ3 |
| IGLJ4 |
| IGLJ5 |
| IGLJ6 |
| IGLJ7 |
| IGLV@ |
| IGLV1-36 |
| IGLV1-40 |
| IGLV1-44 |
| IGLV1-47 |
| IGLV1-50 |
| IGLV1-51 |
| IGLV10-54 |
| IGLV11-55 |
| IGLV2-11 |
| IGLV2-14 |
| IGLV2-18 |
| IGLV2-23 |
| IGLV2-33 |
| IGLV2-8 |
| IGLV3-1 |
| IGLV3-10 |
| IGLV3-12 |
| IGLV3-16 |
| IGLV3-19 |
| IGLV3-21 |
| IGLV3-22 |
| IGLV3-25 |
| IGLV3-27 |
| IGLV3-32 |
| IGLV3-9 |
| IGLV4-3 |
| IGLV4-60 |
| IGLV4-69 |
| IGLV5-37 |
| IGLV5-39 |
| IGLV5-45 |
| IGLV5-48 |
| IGLV5-52 |
| IGLV6-57 |
| IGLV7-43 |
| IGLV7-46 |
| IGLV8-61 |
| IGLV9-49 |
| C3 |
| C5 |
| CCL3L2 |
| CKLF |
| CMA1 |
| CX3CL1 |
| CXCL17 |
| CYR61 |
| EDN1 |
| EDN2 |
| EDN3 |
| FGF10 |
| LECT2 |
| PPBPL1 |
| PROK2 |
| SAA1 |
| SAA2 |
| SBDS |
| SEMA3A |
| SEMA3B |
| SEMA3C |
| SEMA3D |
| SEMA3E |
| SEMA3F |
| SEMA3G |
| SEMA4A |
| SEMA4B |
| SEMA4C |
| SEMA4D |
| SEMA4F |
| SEMA4G |
| SEMA5A |
| SEMA5B |
| SEMA6A |
| SEMA6B |
| SEMA6C |
| SEMA6D |
| SEMA7A |
| SLIT1 |
| SLIT2 |
| TNC |
| TYMP |
| C5AR1 |
| CCR9 |
| CCRL2 |
| CMKLR1 |
| CX3CR1 |
| CXCR3 |
| CXCR5 |
| CXCR7 |
| CYSLTR1 |
| CYSLTR2 |
| DARC |
| EDNRA |
| EDNRB |
| FPR1 |
| FPR2 |
| GPR17 |
| GPR32 |
| GPR33 |
| GPR44 |
| GPR77 |
| IL8RB |
| LTB4R2 |
| PLAUR |
| PLXNA1 |
| PLXNA2 |
| PLXNA3 |
| PLXNA4 |
| PLXNB1 |
| PLXNB2 |
| PLXNB3 |
| PLXNC1 |
| PLXND1 |
| PTAFR |
| ROBO1 |
| ROBO2 |
| RXFP3 |
| XCR1 |
| ADM |
| ADM2 |
| AGRP |
| AGT |
| AMBN |
| AMELX |
| AMH |
| ANGPTL5 |
| ANGPTL7 |
| APLN |
| AREG |
| ARMET |
| ARMETL1 |
| ARTN |
| AVP |
| BDNF |
| BMP1 |
| BMP10 |
| BMP15 |
| BMP2 |
| BMP3 |
| BMP4 |
| BMP5 |
| BMP6 |
| BMP7 |
| BMP8A |
| BMP8B |
| BTC |
| C19orf10 |
| CALCA |
| CALCB |
| CAT |
| CCK |
| CD320 |
| CD70 |
| CECR1 |
| CER1 |
| CGA |
| CGB |
| CGB1 |
| CGB2 |
| CGB5 |
| CGB7 |
| CGB8 |
| CHGA |
| CHGB |
| CLCF1 |
| CLEC11A |
| CMTM1 |
| CMTM2 |
| CMTM3 |
| CMTM4 |
| CMTM5 |
| CMTM6 |
| CMTM7 |
| CMTM8 |
| CNTF |
| CORT |
| CRH |
| CSF1 |
| CSF2 |
| CSF3 |
| CSH1 |
| CSH2 |
| CSHL1 |
| CSPG5 |
| CTF1 |
| CTGF |
| DKK1 |
| EBI3 |
| EGF |
| EPGN |
| EPO |
| EREG |
| ESM1 |
| FAM3B |
| FAM3C |
| FAM3D |
| FGF1 |
| FGF11 |
| FGF12 |
| FGF13 |
| FGF14 |
| FGF16 |
| FGF17 |
| FGF18 |
| FGF19 |
| FGF20 |
| FGF21 |
| FGF22 |
| FGF23 |
| FGF3 |
| FGF4 |
| FGF5 |
| FGF6 |
| FGF7 |
| FGF8 |
| FGF9 |
| FIGF |
| FIGNL2 |
| FLT3LG |
| FSHB |
| GAL |
| GALP |
| GAST |
| GCG |
| GDF1 |
| GDF10 |
| GDF11 |
| GDF2 |
| GDF3 |
| GDF5 |
| GDF6 |
| GDF7 |
| GDF9 |
| GDNF |
| GH1 |
| GH2 |
| GHRH |
| GHRL |
| GIP |
| GKN1 |
| GMFB |
| GMFG |
| GNRH1 |
| GNRH2 |
| GPHA2 |
| GPHB5 |
| GPI |
| GREM1 |
| GREM2 |
| GRP |
| GUCA2A |
| HBEGF |
| HDGF |
| HDGFRP3 |
| IAPP |
| IFNE |
| IFNK |
| IFNW1 |
| IGF1 |
| IGF2 |
| IL11 |
| IL12A |
| IL16 |
| IL17B |
| IL17C |
| IL17D |
| IL17F |
| IL19 |
| IL1F10 |
| IL1F5 |
| IL1F6 |
| IL1F7 |
| IL1F8 |
| IL1F9 |
| IL1RN |
| IL20 |
| IL21 |
| IL23A |
| IL24 |
| IL25 |
| IL26 |
| IL28B |
| IL3 |
| IL31 |
| IL32 |
| IL33 |
| IL34 |
| IL5 |
| IL6ST |
| IL7 |
| IL9 |
| INHA |
| INHBA |
| INHBB |
| INHBC |
| INHBE |
| INS |
| INS-IGF2 |
| INSL3 |
| INSL4 |
| INSL5 |
| INSL6 |
| JAG1 |
| JAG2 |
| KGFLP1 |
| KGFLP2 |
| KITLG |
| KL |
| LACRT |
| LEFTY1 |
| LEFTY2 |
| LHB |
| LIF |
| LRSAM1 |
| LTB |
| LTBP2 |
| LTBP3 |
| LTBP4 |
| MDK |
| MIA |
| MLN |
| MSTN |
| NAMPT |
| NDP |
| NENF |
| NGF |
| NMB |
| NODAL |
| NOV |
| NPFF |
| NPPA |
| NPPB |
| NPPC |
| NPY |
| NRG1 |
| NRG2 |
| NRG3 |
| NRG4 |
| NRTN |
| NTF3 |
| NTF4 |
| NTS |
| NUDT6 |
| OGN |
| OSGIN1 |
| OSM |
| OSTN |
| OXT |
| P11 |
| PDGFA |
| PDGFB |
| PDGFC |
| PDGFD |
| PDGFRL |
| PGF |
| PMCH |
| PNOC |
| POMC |
| PPBPL2 |
| PPY |
| PRL |
| PRLH |
| PROK1 |
| PSPN |
| PTH |
| PTH2 |
| PTHLH |
| PTN |
| PYY |
| QRFP |
| RABEP1 |
| RABEP2 |
| REG1A |
| RETN |
| RETNLB |
| RLN1 |
| RLN2 |
| RLN3 |
| SCG2 |
| SCGB3A1 |
| SCT |
| SCYE1 |
| SECTM1 |
| SLURP1 |
| SPP1 |
| SST |
| STC1 |
| STC2 |
| TAC1 |
| TDGF1 |
| TDGF3 |
| TG |
| TGFA |
| TGFB2 |
| TGFB3 |
| THPO |
| TNFRSF11B |
| TNFSF12 |
| TNFSF13 |
| TNFSF13B |
| TNFSF14 |
| TNFSF15 |
| TNFSF18 |
| TNFSF8 |
| TNFSF9 |
| TOR2A |
| TRH |
| TSHB |
| TSLP |
| TXLNA |
| UCN |
| UCN2 |
| UCN3 |
| UTS2 |
| UTS2D |
| VEGFB |
| VEGFC |
| VGF |
| VIP |
| ACVR1B |
| ACVR1C |
| ACVR2A |
| ACVR2B |
| ACVRL1 |
| ADCYAP1R1 |
| ADIPOR1 |
| ADIPOR2 |
| ADRB1 |
| ADRB2 |
| AGTR1 |
| AGTR2 |
| AMHR2 |
| ANGPT1 |
| ANGPT4 |
| ANGPTL1 |
| ANGPTL2 |
| ANGPTL3 |
| ANGPTL4 |
| ANGPTL6 |
| APLNR |
| AR |
| AVPR1A |
| AVPR1B |
| AVPR2 |
| BMPR1A |
| BMPR1B |
| BMPR2 |
| BRD8 |
| C3AR1 |
| CALCR |
| CALCRL |
| CNTFR |
| CRHR1 |
| CRHR2 |
| CRIM1 |
| CRLF1 |
| CRLF2 |
| CRLF3 |
| CSF1R |
| CSF2RA |
| CSF2RB |
| CSF3R |
| EGFR |
| ENG |
| EPOR |
| ESR1 |
| ESR2 |
| ESRRA |
| ESRRB |
| ESRRG |
| FGFR1 |
| FGFR2 |
| FGFR3 |
| FGFR4 |
| FGFRL1 |
| FLT1 |
| FLT3 |
| FLT4 |
| FSHR |
| GALR2 |
| GALR3 |
| GCGR |
| GHR |
| GHRHR |
| GHSR |
| GIPR |
| GLP1R |
| GLP2R |
| GNRHR |
| GPER |
| HNF4A |
| HNF4G |
| HTR3A |
| HTR3B |
| HTR3C |
| HTR3D |
| HTR3E |
| IFNGR2 |
| IGF1R |
| IGF2R |
| IL10RA |
| IL10RB |
| IL11RA |
| IL11RB |
| IL12RB1 |
| IL12RB2 |
| IL13RA1 |
| IL13RA2 |
| IL15RA |
| IL15RB |
| IL17RA |
| IL17RB |
| IL17RC |
| IL17RD |
| IL17RE |
| IL18R1 |
| IL18RAP |
| IL1R1 |
| IL1R2 |
| IL1RAP |
| IL1RL1 |
| IL1RL2 |
| IL20RA |
| IL20RB |
| IL21R |
| IL22RA1 |
| IL22RA2 |
| IL23R |
| IL27RA |
| IL2RA |
| IL2RB |
| IL2RG |
| IL31RA |
| IL3RA |
| IL4R |
| IL5RA |
| IL6R |
| IL9R |
| INSR |
| KDR |
| LEPR |
| LGR4 |
| LGR5 |
| LGR6 |
| LHCGR |
| LIFR |
| LTBR |
| MC1R |
| MC2R |
| MC3R |
| MC4R |
| MCHR1 |
| MCHR2 |
| MET |
| MLNR |
| MPL |
| MTNR1A |
| MTNR1B |
| NGFR |
| NMBR |
| NPR1 |
| NPR3 |
| NR0B1 |
| NR0B2 |
| NR1D1 |
| NR1D2 |
| NR1H2 |
| NR1H3 |
| NR1H4 |
| NR1I2 |
| NR1I3 |
| NR2C1 |
| NR2C2 |
| NR2E1 |
| NR2E3 |
| NR2F1 |
| NR2F2 |
| NR2F6 |
| NR3C1 |
| NR3C2 |
| NR4A1 |
| NR4A2 |
| NR4A3 |
| NR5A1 |
| NR5A2 |
| NR6A1 |
| NRP1 |
| NRP2 |
| OGFR |
| OPRD1 |
| OPRK1 |
| OPRL1 |
| OPRM1 |
| OSMR |
| OXTR |
| PGR |
| PGRMC2 |
| PPARA |
| PPARD |
| PRLHR |
| PRLR |
| PTGER1 |
| PTGER2 |
| PTGER3 |
| PTGER4 |
| PTGFR |
| PTH1R |
| PTH2R |
| RARA |
| RARB |
| RARG |
| RORA |
| RORB |
| RORC |
| RXFP1 |
| RXFP2 |
| RXRA |
| RXRB |
| RXRG |
| S1PR1 |
| S1PR2 |
| SCTR |
| SDC1 |
| SDC2 |
| SDC3 |
| SDC4 |
| SORT1 |
| SSTR1 |
| SSTR2 |
| SSTR5 |
| ST2 |
| TACR1 |
| TEK |
| TGFBR1 |
| TGFBR2 |
| TGFBR3 |
| THRA |
| THRB |
| TIE1 |
| TNFRSF10C |
| TNFRSF10D |
| TNFRSF11A |
| TNFRSF12A |
| TNFRSF13B |
| TNFRSF13C |
| TNFRSF14 |
| TNFRSF17 |
| TNFRSF18 |
| TNFRSF19 |
| TNFRSF1A |
| TNFRSF1B |
| TNFRSF21 |
| TNFRSF25 |
| TNFRSF4 |
| TNFRSF6B |
| TNFRSF8 |
| TNFRSF9 |
| TRHR |
| TSHR |
| TUBB3 |
| VIPR1 |
| VIPR2 |
| PTPN11 |
| ICAM2 |
| ITGAL |
| ITGB2 |
| PAK1 |
| NCR2 |
| TYROBP |
| LCK |
| FCGR3A |
| FCGR3B |
| LOC652578 |
| NCR1 |
| NCR3 |
| CD247 |
| ZAP70 |
| LCP2 |
| LAT |
| PLCG1 |
| SH3BP2 |
| FYN |
| SHC2 |
| SHC4 |
| SHC3 |
| SHC1 |
| GRB2 |
| SOS1 |
| SOS2 |
| ARAF |
| BRAF |
| RAF1 |
| HCST |
| CD48 |
| CD244 |
| PRKCA |
| PRKCG |
| SH2D1B |
| SH2D1A |
| FAS |
| GZMB |
| PRF1 |
| CASP3 |
| BID |
| CD3D |
| CD3E |
| CD3G |
| PTPRC |
| ITK |
| TEC |
| NCK1 |
| NCK2 |
| GRAP2 |
| PAK2 |
| PAK3 |
| PAK4 |
| PAK6 |
| PAK7 |
| RHOA |
| CDC42 |
| CD28 |
| ICOS |
| MAP3K8 |
| MAP3K14 |
| CTLA4 |
| CBLC |
| CBL |
| CBLB |
| CDK4 |
| RASGRP1 |
| PDK1 |
| PRKCQ |
| TRAC |
| TRAJ1 |
| TRAJ2 |
| TRAJ3 |
| TRAJ4 |
| TRAJ5 |
| TRAJ6 |
| TRAJ7 |
| TRAJ8 |
| TRAJ9 |
| TRAJ10 |
| TRAJ11 |
| TRAJ12 |
| TRAJ13 |
| TRAJ14 |
| TRAJ15 |
| TRAJ16 |
| TRAJ17 |
| TRAJ18 |
| TRAJ19 |
| TRAJ20 |
| TRAJ21 |
| TRAJ22 |
| TRAJ23 |
| TRAJ24 |
| TRAJ25 |
| TRAJ26 |
| TRAJ27 |
| TRAJ28 |
| TRAJ29 |
| TRAJ30 |
| TRAJ31 |
| TRAJ32 |
| TRAJ33 |
| TRAJ34 |
| TRAJ35 |
| TRAJ36 |
| TRAJ37 |
| TRAJ38 |
| TRAJ39 |
| TRAJ40 |
| TRAJ41 |
| TRAJ42 |
| TRAJ43 |
| TRAJ44 |
| TRAJ45 |
| TRAJ46 |
| TRAJ47 |
| TRAJ48 |
| TRAJ49 |
| TRAJ50 |
| TRAJ52 |
| TRAJ53 |
| TRAJ54 |
| TRAJ56 |
| TRAJ57 |
| TRAJ58 |
| TRAJ59 |
| TRAJ61 |
| TRAV1-1 |
| TRAV1-2 |
| TRAV2 |
| TRAV3 |
| TRAV4 |
| TRAV5 |
| TRAV7 |
| TRAV8-1 |
| TRAV8-2 |
| TRAV8-3 |
| TRAV8-4 |
| TRAV8-6 |
| TRAV8-7 |
| TRAV9-1 |
| TRAV9-2 |
| TRAV10 |
| TRAV12-1 |
| TRAV12-2 |
| TRAV12-3 |
| TRAV13-1 |
| TRAV13-2 |
| TRAV14DV4 |
| TRAV16 |
| TRAV17 |
| TRAV18 |
| TRAV19 |
| TRAV20 |
| TRAV21 |
| TRAV22 |
| TRAV23DV6 |
| TRAV24 |
| TRAV25 |
| TRAV26-1 |
| TRAV26-2 |
| TRAV27 |
| TRAV29DV5 |
| TRAV30 |
| TRAV34 |
| TRAV35 |
| TRAV36DV7 |
| TRAV38-1 |
| TRAV38-2DV8 |
| TRAV39 |
| TRAV40 |
| TRAV41 |
| TRBC1 |
| TRBC2 |
| TRBD1 |
| TRBD2 |
| TRBJ1-1 |
| TRBJ1-2 |
| TRBJ1-3 |
| TRBJ1-4 |
| TRBJ1-5 |
| TRBJ1-6 |
| TRBJ2-1 |
| TRBJ2-2 |
| TRBJ2-3 |
| TRBJ2-4 |
| TRBJ2-5 |
| TRBJ2-6 |
| TRBJ2-7 |
| TRBV2 |
| TRBV3-1 |
| TRBV4-1 |
| TRBV4-2 |
| TRBV4-3 |
| TRBV5-1 |
| TRBV5-4 |
| TRBV5-5 |
| TRBV5-6 |
| TRBV5-7 |
| TRBV5-8 |
| TRBV6-1 |
| TRBV6-2 |
| TRBV6-3 |
| TRBV6-4 |
| TRBV6-5 |
| TRBV6-6 |
| TRBV6-7 |
| TRBV6-8 |
| TRBV6-9 |
| TRBV7-2 |
| TRBV7-3 |
| TRBV7-4 |
| TRBV7-6 |
| TRBV7-7 |
| TRBV7-8 |
| TRBV7-9 |
| TRBV9 |
| TRBV10-1 |
| TRBV10-2 |
| TRBV10-3 |
| TRBV11-1 |
| TRBV11-2 |
| TRBV11-3 |
| TRBV12-3 |
| TRBV12-4 |
| TRBV12-5 |
| TRBV13 |
| TRBV14 |
| TRBV15 |
| TRBV16 |
| TRBV17 |
| TRBV18 |
| TRBV19 |
| TRBV20-1 |
| TRBV24-1 |
| TRBV25-1 |
| TRBV27 |
| TRBV28 |
| TRBV29-1 |
| TRBV30 |
| TRDC |
| TRDD1 |
| TRDD2 |
| TRDD3 |
| TRDJ1 |
| TRDJ2 |
| TRDJ3 |
| TRDJ4 |
| TRDV1 |
| TRDV2 |
| TRDV3 |
| TRGV9 |
| TRGV8 |
| TRGV5 |
| TRGV4 |
| TRGV3 |
| TRGV2 |
| TRGJP2 |
| TRGJP1 |
| TRGJP |
| TRGJ2 |
| TRGJ1 |
| TRGC2 |
| TRGC1 |
| TRAV6 |
