# Supplementary material for: A new immune signature for survival prediction and immune checkpoint molecules in lung adenocarcinoma
Source: J Transl Med. 2020 Mar 6;18:118. doi: 10.1186/s12967-020-02286-z (PMC7060601; doi:10.1186/s12967-020-02286-z)
Supplement: Supplementary file 3 — Additional file 3: Table S3. 52 potential survival-related immune genes from three cohorts. [file 12967_2020_2286_MOESM3_ESM.docx]

**Table S3. 52 potential survival-related immune genes from three cohorts.**

| **Gene symbol** |
| --- |
| S100A7 |
| ADM |
| SEMA4B |
| S100A16 |
| IL11RA |
| ARRB1 |
| ADRB1 |
| MIF |
| BMP5 |
| PLAUR |
| GPI |
| PSMD14 |
| RFXAP |
| IL1R2 |
| LCN10 |
| TUBB3 |
| BIRC5 |
| GREM1 |
| CD22 |
| THRA |
| ROBO2 |
| IL20RB |
| KL |
| INHBA |
| GDF10 |
| FURIN |
| PSME3 |
| CD40LG |
| NR0B2 |
| NOX4 |
| RETN |
| SHC3 |
| LAT |
| FGFR2 |
| STC2 |
| CCL17 |
| TMEM173 |
| NMB |
| CIITA |
| STC1 |
| ZAP70 |
| CCR6 |
| S100P |
| PLXNA2 |
| RORA |
| SCGB3A1 |
| HDGF |
| IL33 |
| LIFR |
| CSF2 |
| CD1C |
| PTHLH |
