# Supplementary figures and images for: A new immune signature for survival prediction and immune checkpoint molecules in lung adenocarcinoma
Source: J Transl Med. 2020 Mar 6;18:118. doi: 10.1186/s12967-020-02286-z (PMC7060601; doi:10.1186/s12967-020-02286-z)

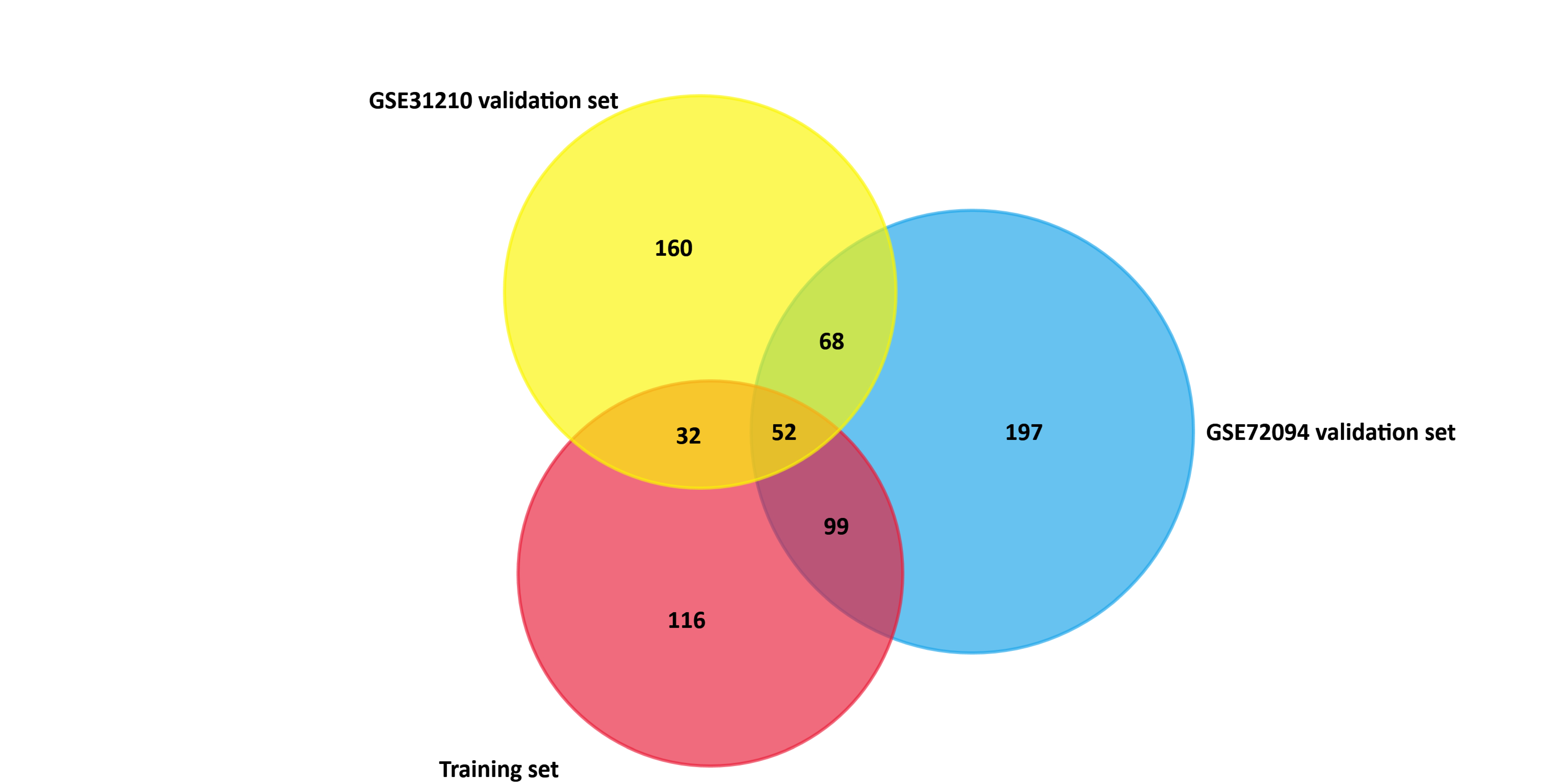

Supplement: Supplementary file 4 — Additional file 4: Figure S1. Venn diagram of the overlapping immune-related genes with the survival using univariate Cox analysis with P < 0.05 from three data sets. [file 12967_2020_2286_MOESM4_ESM.tif]

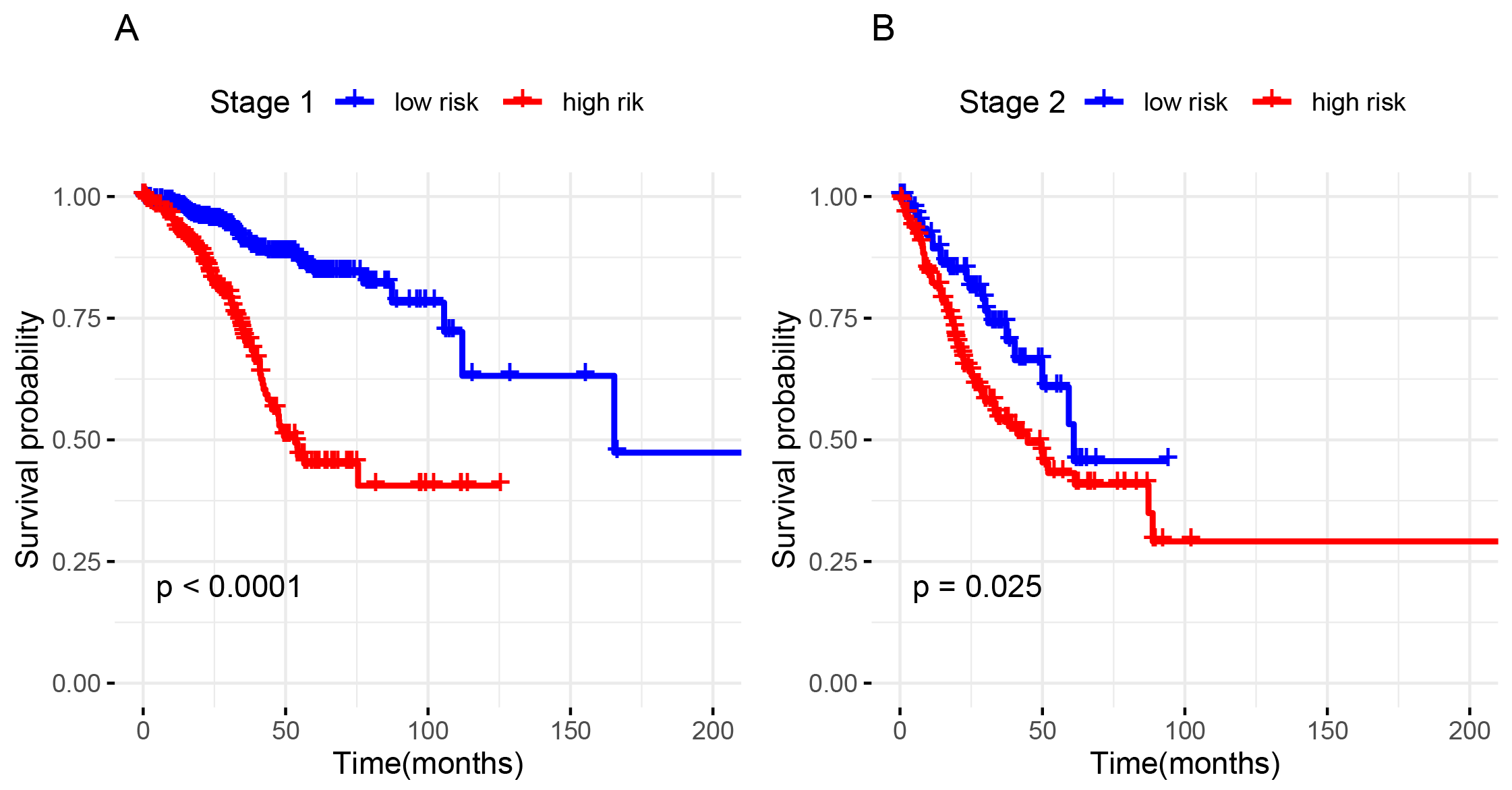

Supplement: Supplementary file 6 — Additional file 6: Figure S2. Kaplan–Meier analyses of our immune signature in stage 1 and stage 2 tumors, including (A) stage 1 lung adenocarcinoma (LUAD) and (B) stage 2 LUAD. [file 12967_2020_2286_MOESM6_ESM.tif]
