# Supplementary material for: A new immune signature for survival prediction and immune checkpoint molecules in lung adenocarcinoma
Source: J Transl Med. 2020 Mar 6;18:118. doi: 10.1186/s12967-020-02286-z (PMC7060601; doi:10.1186/s12967-020-02286-z)
Supplement: Supplementary file 5 — Additional file 5: Table S4. Final 10 immune-related genes in the current model. [file 12967_2020_2286_MOESM5_ESM.docx]

**Table S4. Final 10 immune-related genes in the current model.**

| **Gene symbol** |
| --- |
| FURIN |
| PSMD14 |
| ARRB1 |
| TUBB3 |
| ADM |
| ZAP70 |
| RFXAP |
| SHC3 |
| BMP5 |
| CD40LG |
