# Supplementary material for: A new immune signature for survival prediction and immune checkpoint molecules in lung adenocarcinoma
Source: J Transl Med. 2020 Mar 6;18:118. doi: 10.1186/s12967-020-02286-z (PMC7060601; doi:10.1186/s12967-020-02286-z)
Supplement: Supplementary file 7 — Additional file 7: Table S5. Multivariate Cox analyses of our immune signature in early-stage lung adenocarcinoma (LUAD). [file 12967_2020_2286_MOESM7_ESM.docx]

Table S5. Multivariate Cox analyses of our immune signature in early-stage lung adenocarcinoma (LUAD).

| Variables | Univariate Cox analysis |  | Multivariate Cox analysis |  |
| --- | --- | --- | --- | --- |
|  | Hazard ratio (95% CI) | *P* | Hazard ratio (95% CI) | *P* |
| Immune signature (high- vs. low-risk) | 3.45 (2.57-4.64) | < 0.0001 | 3.93 (2.38-6.5) | < 0.0001 |
| Age (≥65 vs. ＜65 years) | 1.99 (1.50-2.64) | < 0.0001 | 2.05 (1.32-3.19) | 0.001 |
| Gender (male vs. female) | 1.29 (0.98-1.69) | 0.065 |  |  |
| Smoking (yes vs. no) | 2.20 (1.50-3.23) | < 0.0001 | 1.74 (1.03-2.94) | 0.038 |
| KRAS (mutation vs. wild-type) | 2.25 (1.52-3.33) | < 0.0001 | 1.04 (0.65-1.64) | 0.88 |
| EGFR (mutation vs. wild-type) | 0.24 (0.14-0.41) | < 0.0001 | 0.50 (0.27-0.93) | 0.028 |

CI: confidence interval.
